# Bleeding by Bruton Tyrosine Kinase-Inhibitors: Dependency on Drug Type and Disease

**DOI:** 10.3390/cancers13051103

**Published:** 2021-03-04

**Authors:** Philipp von Hundelshausen, Wolfgang Siess

**Affiliations:** 1 Institute for Cardiovascular Prevention, Ludwig-Maximilians University (LMU), 80336 Munich, Germany; Philipp.von_Hundelshausen@med.uni-muenchen.de; 2 German Center for Cardiovascular Research (DZHK), Partner Site Munich Heart Alliance, 80336 Munich, Germany

**Keywords:** Btk, platelet, Btk inhibitor, bleeding, Tec, ibrutinib, covalent Btk inhibitor, reversible Btk inhibitor, hemorrhage

## Abstract

**Simple Summary:**

Bruton tyrosine kinase (Btk) is expressed in B-lymphocytes, myeloid cells and platelets. Since the launch of the first in class Btk-inhibitor (BTKi) ibrutinib in 2013, the list of indications and further drug candidates has expanded greatly. BTKi are not only used to treat patients with B-cell malignancies and in development against various autoimmune diseases, but they have been also proposed as novel antithrombotic drugs and been tested in patients with severe COVID-19. The number of BTKi approved or in clinical studies is rapidly increasing. Although X-linked agammaglobulinemia (XLA) patients with Btk deficiency do not show impaired hemostasis, bleeding events are frequently observed upon treatment with many but not all BTKi. This review describes twelve BTKi approved or in clinical trials. By focusing on their pharmacological properties, targeted disease, bleeding side effects and actions on platelets it attempts to clarify the mechanisms underlying bleeding. Moreover, specific platelet function tests in blood are described which will help to estimate the probability of bleeding side effects of newly developed BTKi.

**Abstract:**

Bruton tyrosine kinase (Btk) is expressed in B-lymphocytes, myeloid cells and platelets, and Btk-inhibitors (BTKi) are used to treat patients with B-cell malignancies, developed against autoimmune diseases, have been proposed as novel antithrombotic drugs, and been tested in patients with severe COVID-19. However, mild bleeding is frequent in patients with B-cell malignancies treated with the irreversible BTKi ibrutinib and the recently approved 2nd generation BTKi acalabrutinib, zanubrutinib and tirabrutinib, and also in volunteers receiving in a phase-1 study the novel irreversible BTKi BI-705564. In contrast, no bleeding has been reported in clinical trials of other BTKi. These include the brain-penetrant irreversible tolebrutinib and evobrutinib (against multiple sclerosis), the irreversible branebrutinib, the reversible BMS-986142 and fenebrutinib (targeting rheumatoid arthritis and lupus erythematodes), and the reversible covalent rilzabrutinib (against pemphigus and immune thrombocytopenia). Remibrutinib, a novel highly selective covalent BTKi, is currently in clinical studies of autoimmune dermatological disorders. This review describes twelve BTKi approved or in clinical trials. By focusing on their pharmacological properties, targeted disease, bleeding side effects and actions on platelets it attempts to clarify the mechanisms underlying bleeding. Specific platelet function tests in blood might help to estimate the probability of bleeding of newly developed BTKi.

## 1. Introduction

Bruton’s tyrosine kinase (Btk), a non-receptor cytoplasmic tyrosine kinase expressed in pre-B cells and B-lymphocytes, plays a central role in B-cell receptor (BCR) signaling, and is crucial for B cell development and proliferation [1]. In addition, Btk is expressed in myeloid cells, and an important component of Fcγ receptor signaling (e.g., FcγRIIa and FcγRIIIa) in monocytes/macrophages and neutrophils, and of FcεRI signaling in mast cells and basophils [2]. Upon Btk activation by Fcγ and Fcε receptor stimulation, downstream signaling leads to the expression of pro-inflammatory cytokines, chemokines, and cell adhesion molecules. Btk-deficient mice and Btk inhibitor treated rodents exhibit reduced disease progression in animal models of human rheumatoid arthritis (RA) and systemic lupus erythematodes (SLE) [3]. Consistently, Btk inhibitors (BTKi) have been developed for the treatment of B cell malignancies and various autoimmune diseases [4].

However, bleeding events are frequent in patients treated for B cell malignancies with BTKi. They are apparently not caused by impairment of the plasmatic coagulation system as shown in a study of non-human primates after oral intake of ibrutinib analogs for 10 days. Plasma clotting times and coagulation-dependent skin bleeding tests were not altered [5]. Instead bleeding has been related to inhibition of Btk which is also expressed in megakaryocytes and platelets [6]. However, patients with X-linked agammaglobulinemia (XLA) who are deficient of Btk do not show an impairment of hemostasis [7]. Thus, BTKi, if exclusively inhibiting Btk, should not increase bleeding. Indeed, BTKi at low Btk-specific concentrations have recently been reported to inhibit atherothrombosis and venous thrombosis in preclinical models without impairing hemostasis. They suppressed human atherosclerotic plaque-induced thrombus formation, mainly by inhibiting platelet glycoprotein VI (GPVI)-signaling [8,9], and venous thrombosis by inhibiting platelet CLEC-2 signaling [10,11]. They might also be effective in suppressing thrombo-inflammation in diseases such as COVID-19 by inhibiting platelet CLEC-2 and FcγRIIa signaling [12,13,14]. Indeed, in a retrospective study ibrutinib protected patients with Waldenström macroglobulinemia from COVID-19 associated lung injury [15], and a prospective clinical case series showed remarkable efficacy of the irreversible BTKi acalabrutinib in patients with severe COVID-19 [16]. However, in the subsequent open-label randomized CALAVI phase II trial acalabrutinib treatment of hospitalized patients on top of best supportive care did not increase the proportion of patients who remained alive and free of respiratory failure [17].

In this comprehensive review, twelve BTKi either approved or in clinical trials are described with particular focus on their Btk-selectivity, pharmacokinetic (PK)- and pharmacodynamics (PD)- properties, targeted disease, bleeding side effects and actions on platelets (Table 1). The aim is to provide insights into the possible mechanisms leading to hemorrhage after BTKi treatment. Platelet function tests in blood are discussed which will help to estimate the probability of bleeding side effects instigated by BTKi application.

## 2. Role of Btk in Platelet Signaling and Platelet Effects of BTKi

**Figure 1:**
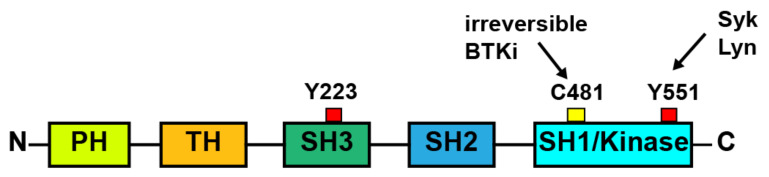
**Schematic representation of the domain-structure of Btk**. PH, pleckstrin homology; TH, Tec homology; SH, Src homology; the SH1 domain is identical to the kinase domain. Y223, autophosphorylation site.

Btk is a member of the cytoplasmic Tec family of tyrosine kinases which comprises also Tec, Bmx (both most homologous to Btk), Itk and Txk/Rlk. Btk carries a pleckstrin homology (PH), a Tec homology, a Src homology 3 (SH3), a SH2, and a kinase domain (Figure 1).

Btk in platelets is involved in GPVI activation by collagen and GPIb activation by VWF [9]. Btk is also critical in mediating FcγRIIa-mediated platelet activation by IgG-containing immune complexes and CLEC-2 activation by podoplanin [10,13]. Btk does not play a role in G-protein coupled receptor stimulated platelet activation by thrombin, thromboxane A2 or ADP. Interestingly, although Btk is activated by fibrinogen ligation of the αIIbβ3 integrin, it does not play a functional role in signaling of this integrin [9].

**Figure 2:**
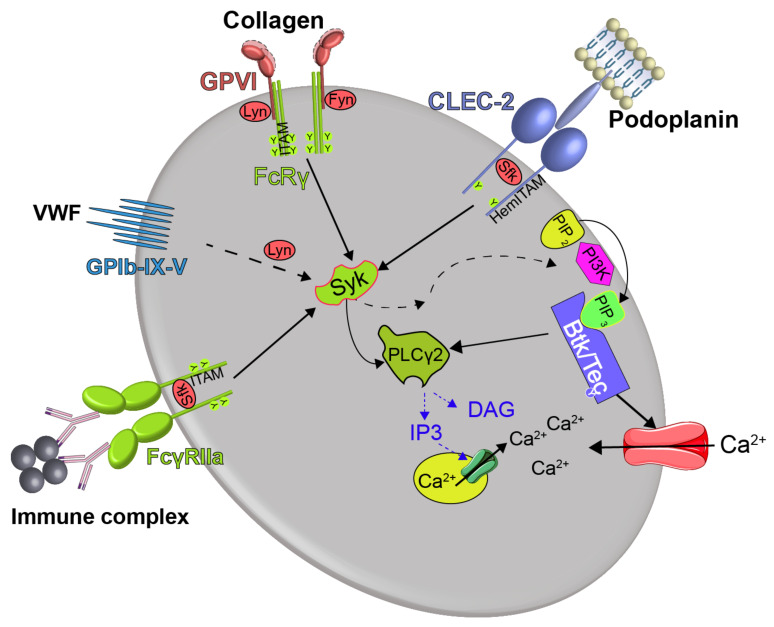
**Btk is activated by platelet glycoprotein receptors and transmits signals to increase cytosolic calcium**. The signaling pathways of platelet glycoprotein receptors that are coupled to Btk are depicted. Solid arrows indicate direct interaction, dashed arrows indicate indirect activation. The homologous kinase Tec is activated through the same pathways but plays a functional role only after GPVI activation. For details see text. CLEC-2, C-type lectin domain family 2; DAG, 1,2-diacylglycerol; FcγRIIa, Fc fragment of IgG low affinity IIa receptor; FcRγ, Fc receptor gamma-chain; GPVI, glycoprotein VI; GPIb-V-IX, glycoproteins Ib,V,IX; hemITAM, single-copy tyrosine-based activation motif; IP3, inositol 1,4,5 triphosphate; ITAM, immunoreceptor tyrosine-based activation motif; PLC2γ2, phospholipase Cγ2; PI3-kinase, phosphatidylinositol 3-kinase; PIP2, phosphatidylinositol(4,5)-bisphosphate; PIP3, phosphatidylinositol(3,4,5)-trisphosphate; SFK, Src family kinases; Syk, spleen tyrosine kinase, VWF, von Willebrand factor.

Btk phosphorylation occurs downstream of activation of GPVI, GPIb, FcγRIIa, and CLEC-2 (Figure 2). The signaling cascades after ligation of these receptors show striking similarities [18,19,20,21]. Activation of the Src family kinases Lyn and Fyn leads via phosphorylation of ITAM (immunoreceptor tyrosine-based activation motif; after GPVI and FcγRIIa stimulation) and hemi-ITAM (after CLEC-2 ligation) to the binding and activation of the tyrosine kinase Syk which phosphorylates the adapter protein LAT. This initiates the formation of a signaling complex comprising further adapter proteins and providing docking sites for phosphatidylinositol (PI) 3-kinase and phospholipase Cγ2 (PLCγ2) [18]. PI3-kinase activation increases membrane levels of PI 3,4,5-trisphosphate (PIP3) that binds with high affinity to the PH-domain of Btk thereby leading to its translocation to the plasma membrane and complex conformational changes of the enzyme also involving the SH2 domain [22,23]. Lyn and Syk then phosphorylate Btk at Y-551 in the kinase domain, and subsequent autophosphorylation at Y-223 in the SH3 domain completes the activation of Btk [18,19,22,24,25] (Figure 1). Y-223 phosphorylation is decisive for kinase activity of Btk. Active Btk participates in the tyrosine phosphorylation and activation of the effector protein PLCγ2 [24,26]. This increases cytosolic Ca^2+^ and activates protein kinase C, the two main downstream signals for platelet activation [27]. Btk also increases Ca^2+^ entry in platelets, independently of PLCγ2 activation [28]. Thus, Btk plays a central role in raising cytosolic Ca^2+^ required for platelet aggregation and secretion.

It has been observed that Btk can play an adapter role independent of its enzymatic activity. A kinase-inactive (K430E) mutant of Btk could reconstitute BCR-induced Ca^2+^ mobilization and ERK/MAPK activation, but not PLCγ2 phosphorylation in Btk-deficient DT40 lymphoma cells [29]. It was suggested that Btk might stabilize the PLCγ2 complex and that this process and not phosphorylation is essential for PLCγ2 activation. However, reconstitution of calcium mobilization in Btk-deficient DT40 cells by a distinct inactive Btk mutant (R525Q) was not observed in another study [30]. Interestingly, in DT40 cells transfected with GPVI and FcRγ and reconstituted with WT or kinase inactive Btk, similar NFAT signaling was observed in response to collagen [31]. It is, however, doubtful whether the observations that Btk can function independently of its enzymatic activity in transfected cells can be translated to platelets. The responses of human and mouse platelets lacking Btk (including its adapter domains) are not more inhibited than the responses of platelets treated with Btk-specific concentrations of BTKi (see Section 2.1, Section 2.2, Section 2.3, Section 2.4 and Section 3).

Tec is also expressed in platelets, although at 20-and 10-times lower levels than Btk as determined in mouse and human platelets, respectively [32,33]. A human platelet contains estimated 1300 protein copies of Tec whereas Btk is expressed at 11,100 copies [33]. Tec is activated after stimulation of platelet GPVI and FcγRIIa [34,35], and the pathway of Tec activation has been reported to be similar to that of Btk [19].

### 2.1. Role of Btk in GPVI Signaling

After GPVI-mediated platelet stimulation by collagen, both Btk and Tec support PLCγ2 activation. As shown in Btk-deficient human platelets from patients with XLA and Btk-deficient mouse platelets, Btk is required for platelet activation only after low-degree GPVI activation [35,36], whereas Tec compensates for the absence of Btk in GPVI signaling and restores platelet reactivity to high concentrations of collagen- related peptide (CRP) or collagen [35].

In accordance with the role of Btk in GPVI signaling, collagen- or CRP- stimulated platelet aggregation and secretion was inhibited by BTKi as studied in washed platelets, PRP or blood. This was found for the irreversible BTKi ibrutinib, acalabrutinib, zanubrutinib, tirabrutinib, evobrutinib and the reversible BTKi fenebrutinib in vitro or after BTKi treatment of patients (ibrutinib, acalabrutinib), or volunteers (ibrutinib) ex vivo [8,13,31,37,38,39,40,41].

In these and the following studies platelet aggregation in blood had been measured mainly by multiple electrode aggregometry (MEA) using hirudin-anticoagulated blood [42] according to a modified protocol to minimize spontaneous platelet aggregation [43].

Studies with washed platelets, PRP and blood showed that low concentrations of ibrutinib and other Btk inhibitors effectively prevented the low degree of GPVI-dependent static platelet aggregation induced by low collagen and CRP concentrations and saturating plaque concentrations [5,9,31,39,41]. Btk and not Tec inhibition correlated with the suppression of PRP aggregation stimulated by half-maximal collagen concentration in a study using 12 different Btk inhibitors including the reversible BTKi RN486 that does not inhibit Tec [40]. Additionally, the reversible BTKi fenebrutinib which lacks Tec inhibition suppressed GPVI-dependent static platelet aggregation in blood only after low, but not high collagen concentrations [13]. In washed platelets low concentrations of ibrutinib and acalabrutinib suppressed CRP-stimulated platelet Btk-Y223 phosphorylation [31,38,39] (but not Tec phosphorylation) that correlated with inhibition of platelet aggregation stimulated by low CRP concentrations [38,39]. Higher CRP concentrations overcame inhibition of aggregation in washed platelet suspensions by ibrutinib and acalabrutinib despite still complete suppression of Btk activation [31,39], but a significant delay was observed [31]. Additionally, high CRP concentrations reversed inhibition of aggregation of PRP in some patients treated with ibrutinib and acalabrutinib [39]. Increasing the collagen concentrations surmounted also the inhibition of platelet aggregation by ibrutinib, zanubrutinib, acalabrutinib or tirabrutinib in blood albeit not completely; inhibition was still significant [41]. Thus, high collagen concentrations can overcome GPVI-inhibition by Btk-specific concentrations of low concentrations of Btk-inhibitors likely due to activation of Tec [35,44]. Indeed higher concentrations of ibrutinib and acalabrutinib also inhibited Tec phosphorylation in CRP-stimulated platelets [31,39] (for details see Section 3.1.2) [31,39]

Together the studies show that irreversible BTKi selectively inhibit Btk in platelets at low concentrations that leads to inhibition of low degree GPVI-mediated platelet activation, that may be a novel strategy to selectively inhibit atherosclerotic plaque- induced thrombus formation (atherothrombosis) [8,9,45,46]. The partial GPVI inhibition resembles the platelet phenotype of patients with XLA and Btk-deficient mice [35,36]. No difference between the irreversible BTKi exists in this regard [8,41]. It is highly unlikely that the inhibition of low degree GPVI-mediated platelet activation affects hemostasis for the following reasons: (a) patients with XLA do not show a bleeding tendency; (b) bleeding time in vitro as measured by the platelet function analyzer (PFA) device [47,48] is not increased by low concentrations of irreversible BTKi [41]; (c) the reversible BTKi fenebrutinib which does not inhibit Tec does not cause bleeding in clinical trials of patients with RA and SLE [49], and even at high concentrations does not prolong closure time as measured by the PFA in vitro [13].

Treatment with therapeutic doses of many irreversible BTKi will bind beside Btk also Tec in platelets and inhibit GPVI signaling completely (see Section 4.3). This is expected to lead only to a mild bleeding tendency, as studies with GPVI-deficient mice or treatment of non-human primates with GPVI-antibodies suggest [50]. However, concomitant treatment with antiplatelet drugs like aspirin or P2Y12 inhibitors may increase the risk for bleeding as supported by findings in GPVI-deficient or ibrutinib-treated mice [51,52] and shown by the clinical experience of patients with CLL or MCL treated with irreversible BTKi (see Section 4.1).

Strong GPVI inhibition such as elicited by anti-GPVI antibodies increases the closure time in the PFA [53]. This device which aspirates citrate-anticoagulated whole blood under constant vacuum from a reservoir through a capillary and a small hole in a membrane filter coated with collagen and epinephrine is used to simulate primary hemostasis [47]. Aspirin intake typically increases bleeding time in this device [54]. Blood incubation with high concentrations of ibrutinib and many other irreversible BTKi have shown to increase closure time in the PFA [13,41] (see Section 3.1 and Table 2).

### 2.2. Role of Btk in CLEC Signaling

The C-type lectin receptor CLEC-2 is expressed at high levels on platelets and activated by podoplanin [18]. CLEC-2 has a cytoplasmic tail containing a hemITAM, and signals similar to GPVI containing an ITAM [18,55]. Mice with a deficiency in CLEC-2 are protected against deep vein thrombosis [11], and CLEC-2 activation by podoplanin plays a role in inflammation-driven murine hepatic thrombosis [56], and possibly in human venous thrombosis [12] whilst it is apparently not involved in primary hemostasis [55]. A recent study demonstrated that Btk is required in platelet CLEC-2 signaling and that low concentrations of ibrutinib and acalabrutinib inhibit CLEC-2 induced aggregation of washed platelets [10]. Thus low doses of ibrutinib or other irreversible BTKi might be also used to prevent deep vein thrombosis [10,46].

### 2.3. Role of Btk in GPIb Signaling 

Btk has been reported to be involved in botrocetin/von Willebrand factor (VWF) signaling through GPIb [21]. Washed platelets from X-linked immunodeficient mice due to mutated Btk did not aggregate in response to botrocetin/VWF. Apparently, Tec could not substitute for Btk in GPIb signaling in mice [21]. No data are available for platelets from patients with XLA.

Ibrutinib inhibits ristocetin-induced GPIb-dependent platelet aggregation in hirudin-anticoagulated blood in vitro and ex vivo in treated patients with CLL or after ingestion by volunteers [8,13,41,57]. Ristocetin-induced platelet aggregation has been proposed as a tool to monitor the bleeding tendency of patients with CLL treated with ibrutinib [57]. Additionally, other irreversible BTKi as well as the reversible BTKi fenebrutinib which lacks Tec inhibition suppressed ristocetin-induced platelet aggregation in hirudin-anticoagulated blood [13,41]. Thus, ristocetin-stimulated VWF/GPIb signaling also in human platelets requires Btk and not Tec. Therefore, it may not be involved in the impairment of hemostasis observed after treatment with irreversible BTKi (see Section 3.1).

In contrast, if measured in citrated PRP, the ristocetin response was found to be preserved in patients treated with ibrutinib [37,58,59] as well after pre-incubation of citrated PRP with ibrutinib in vitro [58]. Additionally, ristocetin-induced aggregation of citrated PRP treated with the reversible BTKi RN486 which is highly Btk selective and does not inhibit Tec was not diminished in citrated PRP [60]. The reason for the difference could be the test system (blood vs. PRP) or the anticoagulation used (hirudin vs. citrate). The extracellular Ca2+ concentrations might affect the involvement of Btk signaling in Ca2+-dependent platelet thromboxane formation and ADP secretion known to be critical for bocotrecin- or ristocetin-induced platelet aggregation [21,61]. In hirudin-anticoagulated blood, Btk-regulated Ca2+ entry is not perturbed and is efficiently inhibited by BTKi, whereas in citrated PRP with low extracellular Ca2+ concentrations, cytosolic Ca2+ increase depends mainly on PLC activation and subsequent IP3-mediated mobilization from intracellular stores [27]. Under the latter conditions GPIb-induced Syk and not Btk activation might mainly drive PLCγ2 activation and the subsequent cytosolic Ca^2+^-rise (Figure 2).

Pre-incubation (30 min) of heparinized blood from healthy donors with ibrutinib (0.5 µM) decreased the firm platelet adhesion onto immobilized VWF under arterial flow (400/s) [38]. Moreover, platelets from patients with CLL treated with ibrutinib, who had bleeding symptoms (n = 3), hardly adhered onto VWF under flow compared with patients with no bleeding symptoms (n = 3). Although these results are suggestive of a clinical relevance of Btk-inhibitors interfering with VWF/GPIb signaling, there might be alternative explanations for these observations (thrombocytopenia, Src-kinase inhibition by ibrutinib).

Inhibition of VWF/GPIb signaling by BTKi might in addition to inhibition of GPVI signaling be beneficial in reducing atherothrombosis [8]. New aptamers antagonising VWF are being developed to inhibit platelet activation in stroke and acute coronary syndrome [50,62,63] 

### 2.4. Role of Btk in FcγRIIa Signaling

IgG-containing immune complexes, as formed in heparin-induced thrombocytopenia type II (HIT), activate the platelet Fc-receptor FcγRIIa. Increased Btk and Tec phosphorylation has been demonstrated in human platelets upon FcγRIIa activation [34], and using a panel of BTKi including the Btk-selective fenebrutinib, it was shown that Btk and not Tec mediates FcγRIIa-stimulated platelet responses (aggregation, secretion, P-selectin expression, platelet-neutrophil complex formation) [13]. Thus, BTKi might be a novel strategy to inhibit HIT [13]. 

Platelet-FcγRIIa is not only crucial for the pathogenesis of HIT, but may also play a role in immune thrombocytopenia (ITP) [64], an autoimmune disease characterized by autoantibody production by B cells directed mainly against GPIb/GPIX and αIIbβ3-integrin [65]. Platelet-bound antibodies are recognized by Fc receptors on phagocytes, leading to phagocytosis and destruction of platelets, especially in the spleen [65,66]. Additionally, plasma of patients with ITP enhances platelet GPIb shedding that was shown to be dependent on platelet FcγRIIa [67].

## 3. Btk Inhibitors (BTKi)

**Figure 3:**
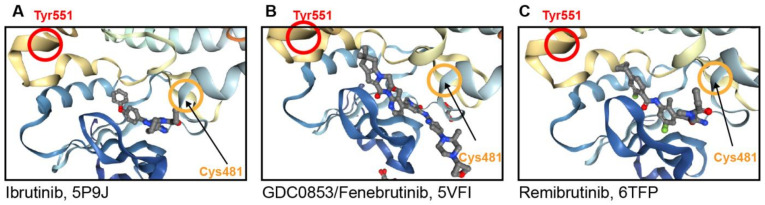
**Crystal structures of Btk in complex with inhibitors**. Secondary structure cartoons of the catalytic domain of Btk in complex with (**A**) Ibrutinib (PDB entry code 5P9J), (**B**) Fenebrutinib (PDB entry code 5VFI) and (**C**) Remibrutinib (PDB entry code 6TFP). Highlighted are Cys481 (orange circle) and Tyr551 (red circle). Snap shots were visualized using the web-based NGL viewer.

All BTKi are orally applied, either as a single dose (QD) or twice (BID) daily. The currently predominant BTKi bind covalently and irreversibly to Cys-481 in the ATP binding site of Btk (Figure 3A). By containing electrophiles, generally acrylamide or related acceptors, the BTKi alkylate Cys-481. These include ibrutinib and the 2nd generation BTKi acalabrutinib, zanubrutinib and tirabrutinib which are approved for the therapy of B-cell malignancies, the brain penetrant evobrutinib and tolebrutinib, both in clinical trials of multiple sclerosis, and branebrutinib, currently in a phase 2 study of various autoimmune diseases (Table 1).

There are several advantages of irreversible covalently BTKi. As compared to reversible inhibitors, their biochemical efficiency for target disruption is increased, they show sustained PD effects which are much longer than the PK of the compounds, and they reach in target cells complete Btk occupancy that is required for clinical efficacy, especially in B-cell malignancies. By inhibiting newly synthesized Btk they achieve to cover Btk more continuously than reversible BTKi. Once the drug is eliminated from systemic circulation, the duration of Btk inhibition becomes independent from drug exposure and is determined by the turnover of the inactive drug/protein complex by synthesis of new Btk protein.

The dependency on covalent binding to Cys-481 poses, however, a problem for the selectivity of the irreversible BTKi, since there are 9 other human kinases with a cysteine residue at a similar position within the ATP binding pocket as Btk: the Tec-family kinases (Bmx, Tec, Txk, Itk), EGFR, Erb2, Erb4, Jak3, and Blk. Indeed ibrutinib, the first BTKi approved, shows more or less strong inhibition of these kinases. Additionally, the second generation irreversible BTKi although more selective than ibrutinib inhibit some of these kinases. All of these BTKi show Tec inhibition in biochemical assays (Table 1).

Furthermore, due to their ATP like binding mode, these compounds can also bind non-covalently to and inhibit even more kinases. This has been observed for ibrutinib which can inhibit various Src-family kinases that are important in platelet signaling. A more remote concern with covalent inhibition is the potential for idiosyncratic adverse drug reactions (IADRs), which are characterized as immunogenicity of a protein adduct (haptenization) leading to an allergic response or drug hypersensitivity reaction. To reduce potential off-target effects of irreversible BTKi, the ideal BTKi should exhibit fast absorption, high exposure, and rapid Btk occupancy in target cells combined with fast elimination (short half-life). This has been best achieved with branebrutinib.

An early observation was the development of resistance to ibrutinib treatment in some patients with B-cell malignancies that was caused by Cys-481 and Thr-474 Btk mutations which arise spontaneously in the malignant cells during ibrutinib therapy. This has led to the development of reversible noncovalent BTK inhibitors with the goal to overcome these Btk mutations. Indeed, reversible BTKi such as fenebrutinib were able to inhibit Btk Cys-481 and Thr-474 mutants with similar potency as wild type Btk [68,69]. A disadvantage of reversible inhibitors is their requirement of continuous systemic drug exposure over the entire dosing interval in order to maintain a high degree of target inhibition.

Reversible BTKi include vecabrutinib, LOXO-305, BMS-986142 and fenebrutinib. They inhibit Btk in the presence of Cys-481 mutations, and some of these BTKi had been in clinical trials of CLL [70]. The vecabrutinib trial has, however, been stopped because of insufficient evidence of activity in BTKi- resistant B-cell malignancies [71]. Additionally, the small phase 1 study with fenebrutinib in patients with B-cell malignancies was prematurely halted [69]. The reason for the negative results in the trials of B-cell malignancies with the reversible BTKi might be their lack to achieve the continuous high coverage of Btk required for clinical efficacy.

Reversible BTKi such as BMS-986142 and fenebrutinib have been advanced into trials of autoimmune diseases (Table 1, Section 3.3.1 and Section 3.3.2). Of particular interest is the reversible covalent BTKi rilzabrutinib, which is in clinical trials of ITP and pemphigus (Table 1, Section 3.4).

Special consideration deserves the novel selective irreversible BTKi remibrutinib, which was obtained by modification of reversible BTKi targeting an inactive conformation of Btk (Table 1, Section 3.5).

The in vitro pharmacology data (IC50, Btk selectivity) of the various BTKi show differences (see Section 3.1, Section 3.2, Section 3.3, Section 3.4 and Section 3.5). This is likely due to the different properties of the BTKi, although different assay conditions could also play a role. Of note, several studies comparing different BTKi in their assays mostly confirmed the different potencies and Btk selectivity found in other studies. The BTKi show higher IC50 values for inhibition of Btk-mediated functions in cell assays than for the inhibition of Btk in vitro. This can be explained by the plasma binding of the drugs, their requirement to cross the cell membranes for Btk binding and the high ATP concentration of the cellular environment. Irreversible BTKi require longer incubation times than reversible BTKi to inhibit Btk in cells. In general, the different potencies of BTKi found in vitro assays correlate well with their different potencies to inhibit Btk in cells including platelets. An interesting exception seems to be evobrutinib (see Section 3.2.1).

### 3.1. Irreversible Covalent BTKi

#### 3.1.1. Ibrutinib

Ibrutinib was the first BTKi to be approved (in 2013). It is approved for the treatment of various B cell malignancies, i.e., chronic lymphocytic leukemia (CLL)/small lymphocytic lymphoma, mantle cell lymphoma (MCL), Waldenström’s macroglobulinemia, and marginal zone lymphoma (MZL) [72]. Recently, based on its additional inhibition of the Tec-family kinase Itk (interleukin-2 inducible kinase) which is involved in proximal T-cell receptor signaling, ibrutinib was shown to be effective for the treatment of steroid-insensitive chronic graft versus host disease (cGVHD) [73] and obtained approval also for treatment of this disease [72].

The dose for MCL is 560 mg QD, for the other diseases 420 mg QD.

The most common adverse reactions (≥30%) in patients with B-cell malignancies treated with ibrutinib, are diarrhea, fatigue, musculoskeletal pain, neutropenia, rash, anemia, thrombocytopenia and bruising [72]. The most common adverse reactions (≥20%) in patients with cGVHD included also bruising (40%), thrombocytopenia (33%) and hemorrhage (26%) [74]. It is likely that some of the adverse effects of ibrutinib are due to its off-target activity. For example, skin rash and diarrhea had been related to EGFR inhibition.

Due to its low bioavailability, high doses (420 mg/560 mg daily) of ibrutinib are required to achieve effective Btk target occupancy [75]. Following complete intestinal absorption ibrutinib undergoes in enterocytes and hepatocytes extensive oxidative metabolism by cytochrome P450 3A (Cyp3A) [76]. Co-administration with the CYP3A inhibitor ketoconazole increases drastically the ibrutinib exposure [77]. The fraction escaping the gut metabolism under fed conditions is about 50%, and the fraction escaping hepatic extraction is 16%. The systemic bioavailabilty in human is 3.9% in the fasting state, and 8.4% under fed condition [76]. Intestinal but not hepatic Cyp3A is irreversibly blocked by furanocoumarins present in the grapefruit juice [76]. Grapefruit intake together with food before ibrutinib administration doubles thereby its bioavailabilty in humans and reaches 15.9% [76].

PK studies in patients with B-cell malignancies showed 2 h after ibrutinib intake of 420 mg QD and 560 mg QD peak plasma concentrations of 0.31 µM and 0.37 µM, respectively [75]. After 560 mg QD of ibrutinib full Btk occupancy (>95%) in peripheral blood mononuclear cells (PBMC) was reached over 24 h. Plasma concentrations subsequently declined rapidly with a mean t ½ of 2–3 h [75].

Ibrutinib very potently inhibits Btk. The IC50 values for ibrutinib reported in various studies using different assays were between 0.13 nM and 1.3 nM, [40,78,79]. Ibrutinib at higher concentrations also binds covalently to the other Tec-family kinases Tec, Bmx, and Itk, and kinases with a similarly accessible cysteine residue such as the EGF-receptor (EGFR) and the tyrosine kinase Jak3 [78,79]. Additionally, ibrutinib has a promiscuous hinge binding moiety that enables the molecule to reversibly bind to kinases that lack the cysteine residue such as the Src family kinases (Src, Lyn, Fyn, and Yes) [79]. These are also expressed in platelets and play an important role in signaling through GPVI, GPIb and the integrin αIIbβ3 [80].

In vitro and ex vivo effects of ibrutinib on platelets have been reported in several studies. Ibrutinib potently inhibited in vitro CRP- or collagen-activated aggregation in PRP and blood [8,9,31,39,41]. Ibrutinib pre-incubated for 60 min with blood inhibited platelet aggregation induced by low collagen concentrations with an IC50 value of 0.025 µM which was well below the therapeutic concentrations of ibrutinib [41] (Table 2). In another study a higher IC50 of 0.35 µM was found for inhibition of aggregation of PRP pre-incubated for 15–45 min and stimulated with half-maximal collagen concentrations [40].

In washed platelets stimulated with CRP, ibrutinib inhibited Btk-Y223 autophosphorylation in two studies with IC50 values of 63 nM and 23 nM, respectively [31,39]. Inhibition of Btk-Y223 auto-phosphorylation by ibrutinib correlated with inhibition of platelet aggregation stimulated by CRP in one [38] but not in another study [31]. Inhibition of total Tec-phosphorylation required about 6-fold higher ibrutinib concentrations (IC50 368 nM, and 150 nM), respectively [31,39]. However, Tec-phosphorylation data might not be as reliable due to the lack of antibodies specific for the Tec autophosphorylation site (Y-206). Ibrutinib at about 30-fold higher concentrations than required for Btk-inhibition (IC50 = 63 nM) inhibited also Src (IC50 2 µM) and Lyn (IC50 1.5 µM) phosphorylation stimulated by CRP in washed platelets [39] confirming results of a previous study [38].

Under arterial flow conditions, ibrutinib (0.2 µM, 0.5 µM) incubated with blood in vitro did not inhibit platelet adhesion and platelet aggregate formation onto collagen [8]. With blood from patients with CLL treated with ibrutinib a significant delay of platelet aggregate formation (similar to ibrutinib 1 µM incubated with blood) was observed, but maximal platelet coverage was not different compared with blood of control patients (containing the same platelet concentration) [8]. No decrease of platelet aggregate formation onto collagen under arterial flow was observed when blood was used from volunteers after intake of 140 mg QD or less for one week [8]. The results were explained by the requirement of integrin α2β1 for platelet adhesion onto collagen under flow whose function was not changed by ibrutinib [8]. The lack of effect of ibrutinib on platelet adhesion onto collagen under flow was also observed in another study [81]. Blood incubated with ibrutinib (1 µM), however, reduced the stability of platelet thrombi formed under flow on collagen, an observation also made with blood from two out of six patients on ibrutinib therapy [39,81]. Thrombus instability may be due to decreased platelet adhesion and aggregate formation on immobilized fibrinogen, possibly caused by inhibition of integrin αIIbβ3 signaling by ibrutinib (1 µM) [41,81].

Ibrutinib also potently inhibited ristocetin- stimulated GPIb mediated platelet aggregation in blood (IC50 85 nM) [8], that was also observed ex vivo in volunteers after intake of low doses and in patients with CLL treated with high doses [8,9,13,57]. Ibrutinib also inhibited in vitro potently FcγRIIa-stimulated platelet aggregation in blood (IC50 80 nM) and ex vivo in human volunteers after intake of a single dose [13]. It increased at 1 µM (but not 0.5 µM) in vitro closure time as measured by the PFA [41] (Table 2).

#### 3.1.2. Acalabrutinib

Acalabrutinib (ACP-196) was the first 2nd generation irreversible covalent BTKi to obtain FDA approval (11/2017). Based on the overall response rate for the treatment of adult patients with MCL who have received at least one prior therapy the approval was accelerated. Later it also obtained approval for the treatment of CLL [82]. Acalabrutinib is a more selective BTKi with improved oral absorption as compared to ibrutinib. The recommended dosage is 200 mg (2 × 100 mg capsules) orally and taken with or without food.

The most common adverse reactions (≥30%) in patients with B-cell malignancies treated with acalabrutinib are headache, diarrhea, fatigue, musculoskeletal pain, neutropenia, anemia, upper respiratory tract infections, thrombocytopenia, and contusions [82,83]. Bleeding events grade 1 and 2 are also frequent (see Section 4.1).

In kinase assays of several studies acalabrutinib was about 3–15 fold less potent than ibrutinib. The reported IC50 values in three studies employing different kinase panels and comparing the two BTKi were for acalabrutinib 2.79 nM, 5.1 nM, 19.7 nM and for ibrutinib 0.29 nM, 1.5 nM, 1.3 nM, respectively [40,78,79]. Inhibition of BCR stimulated CD69 expression on B-cells in human whole blood required for acalabrutinib a 20-fold higher IC50 (198 nM) than ibrutinib (12 nM) [79].

Initially acalabrutinib had been described not to inhibit Tec [78]. Subsequently, however, a study employing four different kinase assay platforms demonstrated Tec-inhibition by acalabrutinib and similar selectivity profiles of ibrutinib and acalabrutinib for inhibition of Btk over Tec [40]. Acalabrutinib, in contrast to ibrutinib, did not inhibit the Tec-family kinases Itk and Txk, EGFR, ERBB2, and Src-kinases (Src, Lyn, Fyn, Yes and Lck) [78,79].

Acalabrutinib was rapidly absorbed and eliminated after oral administration. Mean peak plasma values were reached about 1 h after intake. The mean plasma half-life was approximately 1 h independent of dosage. Mean Cmax after the therapeutic dosage of 100 mg BID of acalabrutinib was 1.78 µM, yielding a continuously high BTK occupancy in PBMC (99% after 4 h of dosing and of 97% before the next dose administration) [78].

In vitro and ex vivo effects of acalabrutinib on platelets have been investigated in several studies. The lower potency of acalabrutinib compared with ibrutinib for Btk inhibition in biochemical kinase assays paralleled its lower potency to inhibit in vitro CRP- or collagen-activated aggregation in washed platelets, PRP, or blood [8,9,31,39,41]. Acalabrutinib was about 15 times less potent than ibrutinib in inhibiting GPVI-mediated platelet aggregation in hirudin-anticoagulated blood [41] (Table 2). Additionally, in washed platelets the IC50 values for inhibition of CRP-induced platelet aggregation by acalabrutinib were in two studies 10- and 20- fold higher than those of ibrutinib [31,39]. Acalabrutinib inhibited also ristocetin-induced platelet aggregation and FcγRIIa-stimulated platelet aggregation in whole blood [13,41] (Table 2) and increased at 5 µM (but not 2 µM) closure time as measured by the PFA [41].

Platelet effects of acalabrutinib were compared with ibrutinib in vitro and in treated patients ex vivo [39]. Low doses of ibrutinib and acalabrutinib which were sufficient to inhibit Btk did not inhibit CRP-stimulated Tec phosphorylation in washed platelets but higher concentrations of both BTKi inhibited Tec phosphorylation, too. Probably due to the lack of an antibody specific for the autophosphorylation site of Tec, dose–response curves showed that low concentrations of ibrutinib and acalabrutinib even increased CRP-stimulated total Tec phosphorylation in platelets [39].

Acalabrutinib even at 10 µM did not show inhibition of Src-kinases. In patients with non-Hodgkin lymphoma (NHL) receiving ibrutinib or acalabrutinib aggregation of PRP in response to collagen and CRP was similarly reduced. However, platelet thrombi formed on collagen under arterial flow were unstable with blood from two out of six patients on ibrutinib but not acalabrutinib therapy. This difference might be explained by the ibrutinib mediated inhibition of Src-kinases that are involved in integrin αIIbβ3 outside-in signaling and platelet adhesion to immobilized fibrinogen [80,81]. In support, incubation of blood with high concentrations of ibrutinib (1 µM) but not acalabrutinib (5 µM) was found to significantly reduce platelet aggregation and adhesion to immobilized fibrinogen at arterial flow [41].

#### 3.1.3. Zanubrutinib

Zanubrutinib (BGB-3111) is an irreversible covalent BTKi and has been approved (11/2019) for the treatment of adult patients with MCL who have received at least one prior therapy [84]. Accelerated approval was given based on overall response rate. Zanubrutinib is a potent and more selective BTKi inhibitor with improved oral absorption and better target occupancy as compared to ibrutinib. The recommended dosage is 160 mg orally twice daily or 320 mg orally once, and absorption is independent of food intake [84].

The most common adverse reactions (≥20%) included neutropenia, thrombocytopenia, bruising, decreased hemoglobin, upper respiratory tract infection, rash, diarrhea and cough [84].

Zanubrutinib was very potent (IC50 0.3 nM in kinase assay) and showed a slightly different binding mode with Btk compared with Ibrutinib (IC50 0.18 nM) [85]. Kinase selectivity as determined in a kinase panel of the compound at 1 μM against 342 human kinases showed in addition to Btk inhibition > 70% inhibition of 12 other kinases. In biochemical assays zanubrutinib potently inhibited other kinases with low IC50 values such as EGFR (2 nM), and the Tec family kinases Bmx (0.62 nM), Tec (2 nM), and Txk (2.95 nM), but not Itk, Lck, and JAK3. In cellular assays inhibition of EGFR and Tec by zanubrutinib was about 10 times less than by ibrutinib.

The PD characterization in mice showed rapid (after 0.5 h) maximal Btk occupancy in both PBMC and spleen after oral administration of 14.5 mg/kg zanubrutinib, which coincided with its plasma Cmax of 4.8 μM [85]. Plasma levels dropped rapidly at 2 h to 0.47 µM und subsequently to undetectable levels, while Btk occupancy at 24 h in PBMC was still 80%, in spleen only 30%.

In a phase 1 study zanubrutinib was rapidly absorbed and reached 2 h after the therapeutic dosage of 320 mg QD a mean plasma Cmax of 1.4 µM which decreased to 0.19 µM at 8 h [86]. The mean half-life of zanubrutinib administered either as 160 mg twice daily or 320 mg once daily was 4 h. In PBMCs, complete (>95%) Btk occupancy was achieved 4h post-dose starting already at doses of 40 mg QD per day. Median Btk occupancy in lymph nodes on day 3 of week 1 was 94% in the 320 mg once-daily group and 100% in the 160 mg twice-daily group.

Direct effects of zanubrutinib on platelets have been reported in several studies. Zanubrutinib inhibited similar to ibrutinib Btk-dependent GPVI-, GPIb and FcγRIIa-stimulated platelet aggregation in blood [13,41], but it was with an IC50 of 0.094 µM 4 times less potent than ibrutinib in inhibiting GPVI-mediated platelet aggregation (Table 2). No increased closure time was detected with 1 µM zanubrutinib [41], higher concentrations were not tested (Table 2).

In contrast, another study comparing ibrutinib and zanubrutinib found large differences of their effects on platelets [87]. By using the same doses of ibrutinib and zanubrutinib to incubate PRP and washed platelets and to treat mice, the authors found that ibrutinib but not zanubrutinib induced platelet receptor shedding of GPIb and integrin αIIbβ3 in mice and humans. The comparison of similar dosages of more (ibrutinib) and less (zanubrutinib) potent BTKi on platelets, has however problems [40]. Moreover, the pharmacokinetic data of treated patients show that the plasma exposure of zanubrutinib (2 × 160 mg) is due to the better absorption around 4.5 times higher than for ibrutinib (420 mg) [86] (Table 2). Of possible clinical relevance, treatment of patients with CLL with ibrutinib (n = 6), but not zanubrutinib (n = 5), appeared to result in a significant reduction in platelet surface expression of GPIb and the integrin αIIbβ3, as well as decreased ex vivo thrombus formation on type I collagen under arterial flow [87]. However, the patient cohorts were small and mismatched in important parameters: 3 of 6 ibrutinib treated patients versus 0 of 5 zanubrutinib patients had platelet counts < 100 × 10^9^/l, and 2 of 6 i patients treated with ibrutinib versus 1 of 5 patients treated with zanubrutinib were on antiplatelet drugs. These differences could have influenced the results. Importantly, the apparent large differences of these two BTKi on platelets found in this study did not translate into a different frequency of low grade bleeding events observed during ibrutinib and zanubrutinib therapy of patients (Table 1) (see Section 4.1).

#### 3.1.4. Tirabrutinib

Tirabrutinib (ONO/GS-4059) is an irreversible covalent BTKi and has been approved in Japan for the treatment of recurrent or refractory primary central nervous system lymphoma (PCNSL) (3/2020), Waldenström’s macroglobulinemia (WM) and lymphoplasmacytic lymphoma (LPL) (8/2020) [88,89].

The recommended dosage of tirabrutinib is 480 mg once daily (6 × 80 mg tablets) taken without food.

Like the other BTKi it is mainly eliminated by liver metabolism. The most common adverse reactions (>10%, mainly grade 1 and 2) based on treatment of 37 patients with PCNSL with 320mg and 480mg tirabrutinib include constipation, lymphopenia, anemia, leukopenia, and urinary tract infection [88,89]. Despite the lack of off-target inhibition of EGF receptors in vitro, skin rash was also a common side effect. Based on the analysis of adverse grade ≥3 events in clinical trials of patients with B-cell malignancies hemorrhage was identified as an important risk of tirabrutinib treatment, since serious bleeding was observed in multiple patients in the Japanese and other clinical studies for which a causal relationship with tirabrutinib could not be ruled out [88] (see also Section 4.1).

Tirabrutinib was in biochemical assays about 10-fold less potent (IC50 6.8 nM) for Btk inhibition than ibrutinib (IC50 0.47 nM). It inhibited similar to ibrutinib also the Tec family kinases Bmx (IC50 6 nM), Tec (IC50 48 nM), und Txk (IC50 92 nM), but in contrast to ibrutinib and zanubrutinib barely the EGF-receptor kinases EGFR, ERBB4 and ERBB2, as well as Itk and JAK3 [90]. Similar results were obtained in another study that reported also the lack of inhibition of Src, Lyn and Lck by tirabrutinib [79].

Tirabrutinib was absorbed with maximal plasma concentrations reached between 2 and 3 h post-dose [91]. Plasma levels of tirabrutinib plasma declined rapidly over the 24 h interval sampling period with mean t1/2 values of 6.5 to 8 h. Therapeutic dosages of 320 mg QD and 480 mg QD reached plasma Cmax of 1.95 µM and 2.36 µM, respectively [40,91].

PD measurements were performed using a novel duplex homogeneous Btk occupancy assay using a biotinylated tirabrutinib probe [92]. Whole blood samples from healthy volunteers and patients with CLL incubated for 2 h with increasing concentrations of tirabrutinib showed at 110 nM a 90% Btk occupancy, but Btk occupancy of bone marrow cells and lymph node tissue from patients with CLL was not determined. Since the maximal plasma concentrations greatly exceed this drug level complete Btk occupancy was considered as likely [92].

Direct effects of tirabrutinib on platelets have been investigated in several studies. Tirabrutinib was 7–10 times less potent than ibrutinib in inhibiting GPVI-mediated platelet aggregation in hirudin-anticoagulated blood [8,41]. Thrombus formation onto collagen under arterial shear rate was significantly reduced at 2 µM tirabrutinib pre- incubated for 60min with blood in one study [93], but not in another study with tirabrutinib 2 µM and 5 µM pre-incubated for 15 min with blood [8]. Tirabrutinib inhibited also ristocetin-induced platelet aggregation and FcγRIIa-stimulated platelet aggregation in whole blood [13,41], and increased at 5 µM (but not 2 µM) in vitro bleeding time as measured by the PFA [41] (Table 2).

In patients with B-cell malignancies treated within a clinical trial with a low dose of tirabrutinib (80 mg/day) for 1 month, a tendency of decreased aggregation following collagen 3.3 μg/mL stimulation was observed compared to pretreatment values [93]. No inhibition of platelet aggregation induced by other platelet stimuli (ADP, TRAP) was found ex vivo similar to the results after tirabrutinib incubation of blood [8].

#### 3.1.5. Branebrutinib

Branebrutinib (BMS-98619) is an irreversible covalent BTKi and is currently in a phase 2 study of rheumatoid arthritis, systemic lupus erythematosus and Sjögren’s syndrome.

Due to its special PK and PD properties branebrutinib is considered as an ideal irreversible BTKi. Following application of a very low dose (1–10 mg QD) it shows a fast and efficient absorption, a rapid rate of systemic Btk occupancy and fast elimination. By reaching a faster rate of Btk inactivation than of drug elimination, off-target interactions are minimized [94].

Branebrutinib was obtained by the conversion of the reversible tetrahydrocarbazole- based series of BTKi (see BMS-986142) into an irreversible inhibitor by incorporation of a simple acrylamide [94,95].

Branebrutinib is very potent (IC50 0.1 nM in kinase assay) and in a panel of 245 kinases was selective for Btk with the exception of some Tec family kinases which required 9, 15 and 90 times higher drug concentrations [94]. These were Tec (IC50 0.9 nM), Bmx (IC50 1.5 nM), and Txk (IC50 9 nM). In human whole blood assays, branebrutinib potently inhibited BCR-stimulated expression of CD69 on B cells with an IC50 of 11 nM. Measurements of Btk inhibition in these assays showed a similar IC50 of 5 nM. In cellular assays of FcγRIIa and FcγRIII stimulated peripheral blood mononuclear cells (PBMC) and BCR-dependent stimulation of B-cells, branebrutinib was equally effective (IC50 0.3 nM) [94].

In pre-clinical PK studies the absolute oral bioavailability of branebrutinib in different species was very high (100% in mice, 74% in rats, 81% in dogs), and the compound was largely cleared within 6 h after dosing [94]. Oral administration of 0.5 mg/kg to BALB/c mice showed already after 4 h 90% Btk inactivation in blood cells. When compared with ibrutinib, branebrutinib was 30-fold more potent at inactivating Btk in mice after a single dose which is explained by the absorption differences of the two BTKi. In a mouse collagen-induced arthritis model of human RA branebrutinib was very effective starting at an oral dose of 0.5 mg/kg QD. This correlated with 97% Btk-inactivation in whole blood. Similar dosages protected the mice in a NZB/W lupus prone mouse model, closely resembling human systemic lupus erythematosus and lupus nephritis [94].

In a double-blind, placebo-controlled, single- and multiple-ascending dose (SAD; MAD) phase I study participants received branebrutinib (SAD: 0.3–30 mg; MAD: 0.3–10 mg) or placebo [96]. Participants in the MAD parts received branebrutinib daily for 14 days and were followed for 14 days post dosing. Branebrutinib was rapidly absorbed and reached plasma Cmax of 162–243 nM 0.5 h after intake of 10 mg in the MAD study. Branebrutinib plasma levels dropped then rapidly with a half-life of 1.2–1.7 h to undetectable levels within 24 h. Btk occupancy in whole blood cells was rapid, with 100% occupancy reached after a single 10 mg dose and was maintained for 24 h. In the MAD study 100% Btk occupancy was maintained with only 3 mg QD. Doses of only ≥1 mg QD branebrutinib providing a Btk occupancy of >90% were projected to be efficacious [96]. Branebrutinib intake for 2 weeks was well tolerated. The AEs were mild/moderate, and bleeding events were not observed. Effects on platelets have not been published so far.

Branebrutinib is currently in a phase 2 study (start January 2020) of rheumatoid arthritis, systemic lupus erythematosus and Sjögren’s syndrome with a projected completion date of June 2022.

#### 3.1.6. BI 705564

BI 705564 is an irreversible, covalent BTKi, being developed for the treatment of SLE/LN, RA and allergic disorders.

BI 705564 inhibits Btk with an IC50 of 0.28 nM, and at 3 μM >80% only 3 other kinases (Bmx, Txk and Tec) in a panel screen of 282 kinases [97]. In a double-blind, placebo-controlled, phase I study (SAD, MAD for 14 days) BI 705564 plasma concentrations were found to peak at 1–4 h, before declining in a biphasic manner with a terminal t1/2 of 10 h to 17 h dependent on the tested doses [97]. As observed for ibrutinib, food increased the BI 705564 exposure. BI 705564 at single doses of 20 mg to 80 mg under fed conditions resulted in maximal plasma concentrations of 20 nM to 35 nM and an average Btk occupancy of ≥85% in PBMC that was maintained for up to 48 h after dose administration. BI 705564 doses between 20 mg and 160 mg showed maximal inhibition of B cell activation of 70–100% at 24 h. Inhibition of B cell activation by BI 705564 correlated with Btk occupancy.

BI 705564 was well tolerated but was associated in the MAD study with mild bleeding-related adverse events (petechiae, hematoma, epistaxis, hematuria) in 7/48 participants. The skin bleeding time (as measured by a modified Ivy method) was increased in 18/48 participants, and the median was slightly increased for the groups receiving doses ≥20 mg at days 7 and 14. In a subgroup receiving 40 mg BI 705564 for 4 weeks 7/8 participants showed an increased bleeding time, and the median values measured at days 22 and 28 were >480 s (the upper limit of normal). Consistently, the PFA closure time using the collagen/epinephrine cartridge was found to be prolonged in all 8 participants of this subgroup [97].

### 3.2. Brain-Penetrant Irreversible Covalent BTKi

#### 3.2.1. Evobrutinib 

Evobrutinib is an irreversible covalent BTKi developed to treat autoimmune diseases. It crosses the blood–brain barrier and has shown clinical efficacy in trials of MS.

Evobrutinib inhibits Btk in vitro less potently than other BTKi (IC50 values in two studies 58 nM and 38 nM [79,98]. Kinase screening panel assays showed besides Btk inhibition (90%) a similar inhibition of Bmx (93%) and Tec (82%) at 1 µM [98]. Similar data were obtained another group using another kinase screen panel [79].

Evobrutinib potently inhibited BCR- and Fc receptor–mediated signaling of human B cells and innate immune cells (monocytes, basophils), respectively [99]. Activation of basophil FcεR signaling in human whole blood was inhibited with an IC50 of 1.66 µM, whilst the IC50 values for inhibition of FcγR signaling in the U937 monocytic cell line (78 nM), for B-cell activation in PBMC (18 nM) and cells in whole blood (84 nM) were much lower. PK/PD data in mice showed that a mean BTK occupancy in blood cells of 80% was linked to near-complete disease inhibition in both RA and SLE mouse models. Maximal (>80% after 4 h) and prolonged (24 h) Btk occupancy in blood cells was achieved with 5 mg/kg oral dosing in mice, and the maximal plasma concentrations levels measured at the earliest time point after intake were about 233 nM (100 ng/mL). Plasma concentrations dropped to about 23 nM 6 h after intake [99].

Surprisingly no PK and PD data are available in humans although evobrutinib has completed important clinical trials. Although evobrutinib has demonstrated efficacy in a mouse model of rheumatoid arthritis and a rat model of collagen antibody–induced arthritis, recent results of a phase IIb trial showed that evobrutinib given at dosages of 25 mg QD, 75 mg QD or 50 mg BID for 12 weeks did not improve the response rate in refractory patients with RA [100].

On the other hand evobrutinib (75 mg QD) given in a placebo-controlled trial of multiple sclerosis was effective in significantly reducing the number of gadolinium-enhancing lesions [101]. In addition, a dose-dependent effect in the reduction of the unadjusted annualized relapse rate at week 24 was observed with a rate of 0.08 in the evobrutinib 75 mg-BID group versus 0.37 in the placebo group. The positive effect of evobrutinib on MS was explained by its impact on B-cells and myeloid cells, which play a key role in the pathophysiology of MS, and by its crossing of the blood–brain barrier apparently achieving a high Btk occupancy in the CNS.

Effects of evobrutinib on platelets have been investigated in two studies. Consistent with its higher IC50 for Btk inhibition in biochemical assays, evobrutinib was the least potent of the BTKi studied (about 15- and 50-times less potent than ibrutinib) in inhibiting Btk-dependent platelet aggregation in blood stimulated by FcγRIIa and low collagen concentrations, respectively (Table 2) [13,41]. It also inhibited ristocetin stimulated GPIb-mediated platelet aggregation in blood [41]. Since the therapeutic plasma concentrations are not known, it is unclear, if evobrutinib might inhibit platelets in vivo. Evobrutinib at 10 µM evobrutinib did not increase bleeding time in vitro [41], that might be explained by its 20-fold higher kinase selectivity for Btk over Tec [98].

Evobrutinib taken so far by ≥1200 patients up to 2 years was reported to be safe, and no bleeding has been reported, also not in the clinical trials [102].

#### 3.2.2. Tolebrutinib

Tolebrutinib (SAR442168, PRN2246) is also an irreversible covalent BTKi which crosses the blood–brain barrier and is in development to treat MS. A recent preclinical report showed that tolebrutinib inhibits the myelin loss in a mouse model of MS-like demyelination [103]. Apparently by inhibiting Btk in microglial cells (considered as the immune cells of the brain) tolebrutinib prevented them from destroying the myelin sheaths.

Indeed, in a short report [104], tolebrutinib bound to Btk in microglia-HMC cells with an IC50 of 0.7 nM. It inhibited B-cell receptor stimulation in whole blood with an IC50 of 10 nM. In a kinase screen across 250 kinases 12 kinases were found to be >90% inhibited at 1 μM [104]. A phase 1 study showed a rapid absorption (Tmax = 1 h) of tolebrutinib, and reached after a maximal dose of 90 mg QD for ten days a maximal plasma concentration of 46 nM (21 ng/mL) and Btk occupancy in PBMC of 93–97%. Tolebrutinib was also detectable in cerebrospinal fluid. The drug was well tolerated except diarrhea which was more frequent in the highest level dose group. Bleeding was not observed. Effects on platelets have not been studied [104].

Studies evaluating tolebrutinib in patients with MS are planned.

### 3.3. Reversible BTKi

#### 3.3.1. BMS-986142

BMS-986142 is a reversible non-covalent BTKi developed to treat autoimmune diseases.

Chemically it is a tetrahydrocarbazole containing single stable atropisomer [105]. It potently inhibited Btk in in vitro kinase assays (IC50 0.5 nM), and BCR-stimulated CD69 expression on B cells in whole blood (IC50 8.4 nM). It also inhibited Tec (IC50 10 nM), and the other Tec-family kinases Itk (IC50 15 nM, Txk (28 nM), Bmk (32 nM) and the Scr-kinase Lck (71 nM). As compared with fenebrutinib, BMS-986142 was less selective in a kinase panel assay, and Tec was inhibited by 98% at 1 µM BMS-986142 [79].

BMS-986142 following oral administration of a single dose (5–900 mg) or multiple doses (25–350 mg, once daily for 14 days) was well tolerated and showed favorable PK and PD characteristics in a phase 1 study in healthy volunteers [106]. Dosages of 75 mg, 200 mg, and 350 mg for 14 days yielded mean plasma Cmax of 281, 592 and 1024 ng/mL, respectively (corresponding to 0.49, 1.03, and 1.78 µM, respectively). PD determinations of BCR-stimulated CD69 expression on B cells in whole blood ex vivo showed that the concentration to inhibit 50% of CD69 expression was 0.145 μM (83 ng/mL). Mean maximal inhibition was close to 100% after a single dosage >100 mg and sustained maximal inhibition over 24 h was observed at 14 days after 350 mg QD. The drug was well tolerated, bleeding did not occur.

Encouraged by these studies a placebo controlled efficacy and safety trial of BMS-986142 was carried out in 508 patients with moderate to severe rheumatoid arthritis. Dosing was designed to provide continuous coverage of the IC50 throughout the dosing interval, as supported by preclinical animal studies [106,107]. The patients were on placebo (n = 75), BMS-986142 100 mg/d (n = 73), BMS-986142 200mg/d (n = 73) or BMS-986142 350mg/d (n = 26) for 12 weeks. The primary outcome did, however, not show clinical improvement after BMS-986142 treatment. The percentage of participants achieving American College of Rheumatology 20% (ACR20) or 70% (ACR70) response at week 12 did not show significant differences between the groups. BMS-986142 was well tolerated, no serious adverse events occurred, and bleeding events were not observed [108].

#### 3.3.2. Fenebrutinib

Fenebrutinib (GDC-0853) is a highly selective, reversible non-covalent BTKi developed to treat autoimmune diseases [79].

It interacts with Btk in a different manner than existing covalent and non-covalent inhibitors. Fenebrutinib was developed from CGI1746, a reversible and highly selective BTKi [109]. CGI1746 was the first compound to bind to an inactive conformation of Btk, in which the regulatory Y551 is rotated and forms a new binding pocket (H3 pocket) [110]. This binding mode offers two advantages: (a) it enhances the selectivity of compounds toward Btk due to the sequence variability around the binding pocket. (b) It prevents the phosphorylation of Tyr551 in the activation loop of Btk by upstream kinases, thus increasing the inhibition of Btk. By optimizing CGI1746 in terms of ADME properties, potency, and safety, while maintaining the specific binding mode, fenebrutinib was obtained. The binding of fenebrutinib to the kinase domain is shown in Figure 3B.

Fenebrutinib potently inhibited Btk in in vitro kinase assays (IC50 0.91 nM) [79], and notably in whole blood B-cell activation and Btk autophosphorylation with very low IC50 values of 8.4 nM and 11 nM, respectively. As compared to the covalent BTKi ibrutinib, tirabrutinib, acalabrutinib, evobrutinib and BMS-986142, fenebrutinib as tested in a screen panel of 287 kinases was the most selective BTKi. By testing biochemically the kinases containing a cysteine in analogous position to Btk Cys-481 fenebrutinib was the only BTKi that did not inhibit Tec [79].

Fenebrutinib showed in SAD and MAD studies of healthy volunteers favorable PK and PD properties [111]. A single dose of 50 mg of fenebrutinib resulted in a maximal plasma concentration of 120 nM 1–2 h after intake, decreasing to 30 nM after 8 h. This inhibited Btk dependent basophil activation and IgM mediated B-cell activation in whole blood by >80% and reduced Btk autophosphorylation in whole blood cells by 75% up to 8 h after intake [111]. However, higher dosages (150 mg QD, 200 mg BID) are required to show efficacy in clinical trials [49]. An intake of 150 mg BID for 14 days by human volunteers showed plasma levels of 0.6 µM decreasing only to 0.22 µM 36 h later without dosing. This was associated with an up to 36 h sustained >95% inhibition of Btk-dependent upregulation of CD63 in basophils in blood and Btk autophosphorylation in whole blood cells [111]. Fenebrutinib intake for 14 days in the MAD study did not show any bleeding events.

An initial small phase 1 study evaluated the safety and efficacy of fenebrutinib in relapsed or refractory non-Hodgkin lymphoma (NHL) or chronic lymphocytic leukemia (CLL) [69]. Fenebrutinib (100, 200, or 400 mg/day) was given to 24 patients (including 6 patients who were positive for the C481S mutation). Common adverse events included thrombocytopenia in 25% of the treated patients. A dose of fenebrutinib was not identified that inhibited Btk to the same continuous degree as observed with irreversible BTKi such as ibrutinib and acalabrutinib, and, although clinical efficacy was observed, the trial was prematurely halted. No bleeding (grade 1 and 2) was reported.

A randomized, double-blind, phase 2 trial of patients with rheumatoid arthritis (n = 578) treated for 12 weeks with fenebrutinib showed clinical efficacy in the groups taking higher dosages (1 × 150 mg/day, 2 × 200 mg/day); increased bleeding events were not reported [49]. Additionally, a recent short report of the results of randomized, placebo-controlled study (n = 420) to evaluate the efficacy, safety, and PD effects of fenebrutinib (1 × 150 mg/day, 2 × 200 mg/day for 48 weeks) in patients with moderate-to-severe systemic lupus erythematosus activity (SLE) did not report bleeding [112]. The primary efficacy endpoint for fenebrutinib was, however, not met despite evidence of strong Btk target and pathway inhibition.

The effects of fenebrutinib on platelets have been studied recently [13]. Fenebrutinib was the most potent BTKi in inhibiting Btk-dependent aggregation in blood (Table 2). A concentration as low as 50 nM inhibited completely platelet aggregation upon FcγRIIa, GPIb and low degree GPVI stimulation [13]. Aggregation on thrombin receptor-activating peptide (TRAP), arachidonic acid (AA), or ADP was not compromised. Even very high concentrations of fenebrutinib (up to 1µM) did not increase closure time in vitro (Table 2). Thus, the platelet phenotype is similar to the observations of BTK-deficient human XLA and mouse X-chromosome-linked immune-deficient (XID) platelets [9].

### 3.4. Rilzabrutinib, a Reversible Covalent BTKi

Rilzabrutinib (PRN1008) is an oral, reversible covalent BTKi in clinical development for the treatment of various autoimmune diseases.

Since it was recognized that a long drug-target residence time rather than affinity for the target drives pharmacodynamic activity and disease efficacy in vivo, Btk inhibitors with a prolonged residence time that form a reversible covalent bond with Cys481 were developed [113]. By utilizing an inverted orientation of the cysteine-reactive cyanoacrylamide electrophile, potent and selective BTKi were found that demonstrated tunable enhanced residence times in biochemical assays (from minutes to 7 days) [113]. This strategy resulted in the development of rilzabrutinib.

Binding of rilzabrutinib to Btk shows a fast association and a very slow dissociation rate. It potently inhibited Btk in vitro with an IC50 of 1.3 nM [114]. Significant inhibition with low biochemical IC50 values was observed for five other kinases that share with Btk the conserved Cys-481 as well as a threonine in the gatekeeper position: the Tec-family kinases Tec (IC50 0.8 nM), Bmx (IC50 1 nM), and Txk (IC50 1.2 nM), the receptor tyrosine kinase ERBB 4 (IC50 11 nM) (a member of the EGF receptor family), and Blk (IC50 6.3 nM) [114].

Oral rilzabrutinib showed in SAD (50–1200 mg QD) and MAD (300 to 900 mg QD for 10 days) phase I studies of healthy volunteers [115] rapid absorption under fasted conditions, with Tmax ranging from a median of 0.5 h at 50 mg to 2.5 h at 600 and 1200 mg. Rilzabrutinib demonstrated a half-life of approximately 3–4 h. The maximal plasma concentrations reached a plateau between 1–2 h after dose intake. Plasma Cmax as measured at 10 days were for the therapeutically used dosages of 300 mg BID 0.32 µM, 450 mg BID 0.36 µM and 600 mg QD 0.45 µM. Btk occupancy in PBMC was closely related to the maximal plasma concentrations and was after these rilzabrutinib doses at day 10 >90%, if measured 4 h after dosing, and decreased to about 55–60% 24 h after dosing. The decay of Btk occupancy by rilzabrutinib was slow (–1.6% per h), exhibiting a ~30–35% reduction between 4 h and 24 h. Rilzabrutinib was safe and well tolerated in all dose regimens.

Rilzabrutinib is currently in clinical trials of pemphigus and ITP. Open-label phase 2 trials of both diseases rilzabrutinib at doses of 200 mg QD, 300 mg BID, 400 mg BID for a median time of 10 weeks showed positive results. In the ITP trial (n = 21), 33% reached the primary endpoint (increase of platelet count > 50,000/µl), and in the pemphigus trial, 60 and 87 percent of patients achieved control of disease activity [116,117]. Phase 3 placebo-controlled trials have been started for both autoimmune diseases. Adverse events with rilzabrutinib in the pemphigus and ITP trials were mild-to-moderate, and included mainly nausea, abdominal distension, and dizziness.

Of note, bleeding was not reported, even not in patients with ITP who had a median platelet count at study entry of 14.173/µl [116], but patients with ITP do not show a high risk of bleeding [66]. In a small report, clinically relevant concentrations of rilzabrutinib apparently showed no effect on human platelets in vitro. Rilzabrutinib (0.3 and 1 µM) added to PRP from healthy volunteers or patients with ITP patients did not show reduced platelet aggregation in response to high collagen concentrations (2.5 and 5 µg/mL) in contrast to a high concentration of ibrutinib (1 µM) [118].

### 3.5. Novel Selective Covalent BTKi: Remibrutinib (LOU064), CHMFL-BTK-01

Since second generation covalent inhibitors including branebrutinib retained a similar Btk binding mode as ibrutinib and still inhibit several Cys-containing kinases, alternative binding modes for irreversible BTKi were searched for [119]. Reversible inhibitors such as CGI1746 and fenebrutinib which bind to an inactive conformation of Btk exhibit a very high selectivity for Btk [79,109]. Starting from the scaffold of CGI1746 and combining it with the insertion of an electrophilic acrylamide which binds covalently Cys-481 led to the discovery of the highly potent and selective irreversible BTKi CHMFL-BTK-01 and remibrutinib [110,120,121].

CHMFL-BTK-01 only bound to Btk (IC50 of 4.7 nM) but not any other kinase in a panel of 468 kinases [121]. Additionally, in biochemical assays it did not affect Bmx, EGFR and Jak3 kinase activity. Since oral CHMFL-BTK-01 did not show absorption, this compound was not further followed up.

Towards the development of remibrutinib prototypes were found which showed an excellent Btk- selectivity including several Cys-containing kinases [119]. Further chemical modifications to optimize Btk-binding and PK and PD properties eventually led to the development of remibrutinib [120].

The atomic details of remibrutinib binding to Btk were revealed by X-ray structure analysis. Specific moieties of the compound bound to the kinase hinge region, to the side-chains of amino acids forming the H3-pocket in the inactive conformation of Btk, and covalently to Cys-481 (Figure 3C) [110,120].

Remibrutinib inhibited in biochemical assays very potently Btk (IC50 1.3 nM) [120]. For the determination of kinase selectivity, binding constants (Kd) in a competition binding assay were measured for a set of Cys-containing kinases instead of IC50 values in biochemical enzyme assays, since they were found to be time-dependent and less valid. Remibrutinib showed very potent affinity to Btk with a subnanomolar Kd of 0.63 nM and with a selectivity of 175-fold over Tec (Kd of 110 nM) and 857 fold over Bmx (Kd of 540 nM). Remibrutinib did not show any binding to Itk, EGFR, ERBB2, ERBB4, and Jak3 up to 10 μM. Remibrutinib was the only covalent inhibitor showing a high selectivity for Btk over Tec. The other irreversible covalent Btk-inhibitors tested (ibrutinib, acalabrutinib, tirabrutinib, evobrutinib, and branebrutinib) showed either no or even less selectivity for Btk over Tec. The affinity of remibrutinib was similarly high to that of branebrutinib, and its affinity for Btk was 3-fold higher than of ibrutinib and by factor >20 higher than for the other BTKi. The high Btk-selectivity of remibrutinib was confirmed by cellular assays including primary human cells. In vitro, remibrutinib showed a dose-dependent kinetic of binding to cellular Btk in human blood, and a Btk occupancy >90% was reached after incubation with 100 nM for 100 min.

PK and PD data are so far available only in animal models (rats, mice, dogs) [120]. Here, oral remibrutinib exhibited a rapid and sustained Btk engagement and fast clearance which limits systemic exposure. Oral remibrutinib (3 mg/kg) reached rapidly (0.3–0.8 h post dose) maximal plasma concentrations (ranging from 19 nM and 148 nM in the three species). It was rapidly eliminated (t1/2 0.5–1 h), mainly due to a high hepatic clearance in mice and dog (90%) and showed a low volume of distribution. In rats, a Btk occupancy of 90% was reached in spleen homogenates 5 h after oral dosage of 1.6 mg/kg. Doses of 3 mg/kg, 10 mg/kg and 30 mg/kg remibrutinib were effective in the rat model of collagen-induced arthritis. Efficacy correlated with Btk-occupancy in the spleen.

Remibrutinib is currently in phase 2 clinical studies for chronic spontaneous urticarial (CSU) and Sjoegren’s syndrome [122].

Based on the reported PD properties in blood, remibrutinib is expected to inhibit Btk-mediated platelet signaling in whole blood with high potency and with a Btk-selectivity similar to fenebrutinib. 

## 4. Bleeding After BTKi Treatment and Underlying Mechanisms

### 4.1. Bleeding Events in Patients with B-Cell Malignancies Treated with Irreversible BTKi

Bleeding events are frequent in patients with B-cell malignancies treated with irreversible BTKi, not only with ibrutinib but also the 2nd generation BTKi acalabrutinib, zanubrutinib and tirabrutinib (Table 1) [123,124]. The % values of patients with bleeding events shown in Table 1 are based on the prescribing information of these BTKi, and are likely to be drug-related. This is important, since many patients with CLL and MCL show bleeding independent of BTKi intake (see below and Section 4.2).

Of 2838 patients who received ibrutinib in 27 clinical trials 39% of patients had grade 1 and 2 bleeding events such as bruising and petechiae [72]. Major hemorrhage (≥ Grade 3, such as intracranial hemorrhage, gastrointestinal bleeding, hematuria, and post procedural hemorrhage) occurred in 4% of patients, with fatalities occurring in 0.4% of patients who received ibrutinib in 27 clinical trials. Additionally, patients with cGVHD showed frequently bruising (40%) and hemorrhage (26%) associated with ibrutinib therapy [72].

An integrated analysis of 15 clinical studies of patients with CLL and MCL (n = 1768) on full dose ibrutinib therapy including 4 randomized clinical trials shows that the interpretation of incidence numbers of minor (grade 1,2) and major (grade ≥ 3) bleeding events attributed to ibrutinib treatment (and possibly other BTKi) requires caution [125]. Minor and major bleeding occurred in 35% and 4.4%, respectively, of patients on ibrutinib, but also in 15% and 2.8%, respectively, in the comparator-treated patients in randomized clinical trials. Of note, use of anticoagulants and/or antiplatelet drugs in this analysis was common (~50% of patients) and had an increased exposure-adjusted relative risk (RR) for major bleeding that was similar in the total ibrutinib-treated population (RR 1.9) and the comparator-treated patients (RR 2.4) in randomized clinical trials [125].

The risk of minor bleeding decreases with continued ibrutinib therapy. Whereas ibrutinib-associated risks of minor bleeding events plateaued by 6 months [126], the cumulative incidence of the first major hemorrhage plateaued much later, at 100 weeks [125]. Early low-grade bleeding was not associated with major hemorrhage [125].

Of 1029 patients with CLL and MCL who received acalabrutinib 22% of patients had bleeding events of any grade but not counting bruising and petechiae [82]. If petechiae are included, 31% of patients with MCL on acalabrutinib had bleeding events [127], and in a long-term follow-up study 42% of patients with CLL treated with acalabrutinib had contusions [83]. Major hemorrhage (≥ Grade 3, such as intracranial hemorrhage, gastrointestinal bleeding, hematuria, and post procedural hemorrhage) occurred in 3% of patients, with fatalities occurring in 0.1% of patients who received acalabrutinib.

In patients with MCL treated with zanubrutinib bleeding events of any grade, including purpura and petechiae, occurred in 50% of patients. Grade 3 or higher bleeding events including intracranial and gastrointestinal hemorrhage, hematuria, and hemothorax have been reported in 2% of patients treated with zanubrutinib monotherapy [84].

Concerning tirabrutinib, the patient cohorts are very small. Bleeding events grade 1 and 2 were observed in two clinical studies of treated patients with MCL (n = 16) and CLL (n = 28). The reported frequency of petechiae and purpura was 38% in the MCL study, and the frequency of all grades of bleeding (mostly bruising, petechiae, purpura) was 36% in the long-term 3 year follow-up CLL study [128,129]. Only one grade 3 bleeding event (3.6%; hematoma) occurred in the CLL study. Thrombocytopenia was reported only in the MCL study (44%). Surprisingly, a phase I study of tirabrutinib treatment (160 mg, 320 mg, or 480 mg QD, or 300 mg BID; mean duration 161 days) of patients (n = 17) with relapsed or refractory B-cell malignancies in Japan reported no bleeding events, thrombocytopenia occurred in only 2 patients [130].

Interestingly, less low grade (grade 1–2) skin bleeding events (n = 3, 6.8%) were reported for patients with primary central nervous system lymphoma (PCNSL) (n = 44) treated with tirabrutinib (320 mg QD, 489 mg QD). However, grade ≥ 3 bleeding events were apparently more frequent (n = 4, 9%) (intracranial hemorrhage, subdural hematoma, hematuria) [88].

Thus, the frequencies and profiles of grade 1 and 2 bleeding side effects during therapy of B-cell malignancies with ibrutinib, acalabrutinib, zanubrutinib and tirabrutinib were similar. As compared with the 2nd generation irreversible BTKi, treatment with ibrutinib shows, however, a higher rate of major (grade ≥ 3) and fatal bleeding.

Since ibrutinib, acalabrutinib, and zanubrutinib intake is associated with atrial fibrillation of all grades in 6.5% (6–16%), 4.1%, and 2% of patients, respectively [82,84,131], patients are often treated with anticoagulants to prevent thromboembolism. For tirabrutinib, atrial fibrillation has not been reported, but the patient numbers in the studies were too small to detect this uncommon adverse event [88,129].

Use of either anticoagulant or antiplatelet agents concomitantly with ibrutinib increases the risk of major hemorrhage. Major bleeding observed in 3.1% of 2838 patients on ibrutinib (without antiplatelet drugs or anticoagulants) was increased by the addition of antiplatelet therapy with or without anticoagulant therapy to 4.4%, and by the addition of anticoagulant therapy with or without antiplatelet therapy to 6.1% [72]. Analogous observations were made for the 2nd generation irreversible BTKi, and, hence, the ibrutinib, acalabrutinib and zanubrutinib prescribing information cautions that the co-administration with antiplatelet or anticoagulant medications may further increase the risk of hemorrhage [72,82,84]. Further it is recommended to consider the benefit-risk of withholding the BTKi 3–7 days pre- and post-surgery depending upon the type of surgery and the risk of bleeding.

The treatment with these BTKi also leads frequently to thrombocytopenia, that may be a risk factor for low grade bleeding in patients with CLL and MCL as shown for ibrutinib [125].

### 4.2. Disease-Related Mechanisms of Bleeding

Patients with B-cell malignancies have an intrinsic increased risk for bleeding based on low platelet counts, coagulation disorders, and other comorbidities possibly affecting vascular permeability [125]. Furthermore, CLL lymphocytes express the ectonucleotidase CD39 degrading extracellular ADP and thus reducing platelet aggregation [132]. This might explain why without ibrutinib treatment primary hemostasis measured with the PFA (epinephrine-collagen cartridge) was prolonged in 25% of patients with CLL [126]. Moreover, inflammation in combination with thrombocytopenia leads to loss of vascular integrity and localized hemorrhage which might contribute to the skin bleeding events (grade 1 and 2) observed during treatment of B-cell malignancies with all irreversible BTKi and ibrutinib therapy of cGVHD [133].

Patients treated with different irreversible BTKi for other diseases have not experienced bleeding, however these BTKi have not yet been tested in larger cohorts. For evobrutinib, given in a placebo-controlled trial to 107 patients with MS either at a dosage of 70 mg QD or 70 mg BID for 24 weeks, not a single bleeding event had been reported [101]. Additionally, in a summary of a trial of rheumatoid arthritis given to 293 patients at dosages of 20 mg QD, 50 mg BID and 75 mg QD for 12 weeks bleeding was not mentioned [100].

Additionally, tolebrutinib, a BTKi candidate for MS, sdid not show bleeding in a phase 1 study of human volunteers taken at increasing doses (7.5–90 mg QD) for 10 days [104].

### 4.3. Platelet-Dependent Mechanisms of Bleeding after Treatment of Patients with B-Cell Malignancies with Irreversible BTKi

The high rate of minor bleeding events was similar after treatment of B-cell malignancies with the various irreversible BTKi. Although the BTKi show markedly different potencies for inhibition of Btk in vitro that correlate well with their potencies for platelet inhibition, their effects on platelet activation in blood and PRP do not differ substantially when their differences in plasma exposure are considered [8,13,40,41]. It is obvious and has been emphasized that it is important to compare the potency (IC50 in the platelet assay) of each drug with the observed clinical plasma drug plasma concentrations [40]. For all irreversible covalent BTKi but not for the reversible BTKi fenebrutinib, prolonging the pre-incubation time with blood increased the inhibitory effects [13,41]. As shown in Table 2, the Btk-dependent platelet aggregation in blood after FcγRIIa stimulation or low degree GPVI stimulation was inhibited by much lower concentrations than the antiproliferative plasma concentrations of clinically approved irreversible BTKi (ibrutinib, acalabrutinib, zanubrutinib, tirabrutinib). It is likely that irreversible BTKi at the high doses required for clinical efficacy will produce off-target effects on platelets.

For example, a plasma concentration of 0.3 µM ibrutinib reached after antiproliferative dosage engages completely cellular Btk in human blood within 75 min [120], and will completely and irreversibly inhibit Btk in anucleate platelets for their life time (8–9 days), since these cells lack de novo enzyme synthesis. It is then also to expect that platelet Btk is inhibited in vivo by intake of very low doses of ibrutinib. Although platelet Btk occupancy in blood after ibrutinib intake has not been measured, it was shown that platelet aggregation stimulated by low collagen concentrations in blood was completely inhibited in vitro by 100 nM ibrutinib [41] and after intake of only 140 mg ibrutinib QD on alternate days for 1 week ex vivo [8], a dosage which is 6–8 times lower than the antiproliferative dosage [8]. Additionally, a single dose of 280 mg ibrutinib caused inhibition of Btk-dependent platelet aggregation in response to FcγRIIa, GPIb and low degree GPVI stimulation that was sustained for 2 days. Suppression of platelet dense granule secretion was even decreased up to 7 days after drug intake [13].

It follows that the antiproliferative dosage of ibrutinib is far higher than needed for platelet Btk inhibition. Moreover, pre-systemic drug exposure of platelets in the portal circulation might be due to the high hepatic extraction of ibrutinib (84%) [76] which is several-fold higher than the systemic exposure and will further contribute to off-target effects on platelets. High intra-platelet drug concentrations will lead to irreversible off-target inhibition of Tec. In addition, even higher intra-platelet ibrutinib levels might cause reversible inhibition of platelet Src-kinases (Lyn, Fyn, Yes) causing impaired integrin αIIbβ3 signaling leading to decreased platelet adhesion to fibrinogen and thrombus instability in treated patients (see Section 3.1.1). This could be related to the higher rate of fatal and grade ≥ 3 bleeding events in patients treated with ibrutinib as compared to 2nd generation irreversible BTKi.

As shown in Table 2 the potency of the various irreversible BTK inhibitors to inhibit Btk-dependent platelet stimulation by GPVI- and FcγRIIa-activation in blood was different, and also the plasma concentrations of the 2nd generation BTKi acalabrutinib, zanubrutinib and tirabrutinib after antiproliferative dosage largely exceed the concentrations needed to inhibit in blood Btk-dependent platelet aggregation. Since all these BTKi demonstrate in biochemical assays off-target inhibition of Tec (see Section 3.1.2, Section 3.1.3 and Section 3.1.4), they might at therapeutic concentrations also cause irreversible Tec-inhibition in platelets that could contribute to the frequent grade 1 and 2 bleeding events observed during treatment of B-cell malignancies. Such an interpretation is also consistent with the results that in vitro an increase of the concentrations of these BTKi increased the in vitro bleeding time as assayed with the PFA [120]. Inhibition of both Btk and Tec will lead to complete GPVI-inhibition [35] as observed after antibody inhibition of GPVI [134] that also increases PFA closure time [53] and is likely to be associated with a latent or minor bleeding tendency [50] (see Section 2.1).

The less frequent grade ≥ 3 bleeding events after therapy with these 2nd generation BTKi as compared to ibrutinib could be related to their lack of inhibition of platelet Src-kinases (see Section 3.1.2, Section 3.1.3 and Section 3.1.4). An additional reason could be the high treatment dosage of ibrutinib, higher than pharmacodynamically required. An ibrutinib dosage of 2.5 mg/kg QD was found to result in >95% Btk occupancy in PBMC, but higher doses (420 mg and 560 mg QD for CLL and MCL, respectively, corresponding to 6 mg/kg and 8 mg/kg QD for a person of 70 kg) were selected for treatment, also based on the safety and tolerability the drug [75]. Recent retrospective and pilot studies show comparable outcomes in patients with CLL on reduced dose (280 mg QD, 140 mg QD) or reduced frequency dosing of ibrutinib [135,136,137,138,139]. In one study even the lowest dose (140 mg/d) was sufficient to occupy >95% of Btk in PBMC [136]. Future studies will show, whether bleeding events are reduced with lower ibrutinib doses. 

Co-administration of an antiplatelet drug or anticoagulant increases the risk of hemorrhage (including bleeding events grade ≥ 3) in patients with B-cell malignancies treated with ibrutinib and 2nd generation BTKi [72,82,84]. This is not surprising, since GPVI-inhibition by anti-GPVI antibodies in combination with aspirin increased the inhibition of platelet and fibrin deposition on collagen in humans in vitro under flow and increased bleeding in mice in vivo [51,140]. Additionally, in a mouse model of inflammatory hemorrhage, ibrutinib, which inhibited GPVI-mediated platelet activation without affecting primary hemostasis, showed prolonged bleeding time only in combination with platelet P2Y12 ADP receptor deficiency [52].

### 4.4. No Bleeding in Clinical Trials with Reversible BTKi 

Treatment of patients with B-cell malignancies with reversible BTKi might not be associated with bleeding as a small phase 1 study with fenebrutinib in B-cell malignancies (n = 24) suggests. In this study only two grade 3 bleeding events (both gastrointestinal hemorrhage) were observed, which were possibly related to the intake of acetylsalicylic acid (ASA) or non- steroidal anti-inflammatory drugs (NSAIDs) by these patients [69].

Patients treated with reversible BTKi for autoimmune diseases do not show bleeding events. Patients with RA treated for 12 weeks with BMS-986142 (see Section 3.3.1) which in kinase assays at 20 fold higher IC50 concentrations also inhibits Tec did not show bleeding events, even in the highest dosage group (350 mg QD, n = 26). This dosage reaches maximal plasma concentrations of 1.78 µM, and a complete Btk-occupancy which was sustained for the dosing interval (see Section 3.3.1). Platelet effects of this potent reversible BTKi have not been studied.

Similarly, a phase 2 trial of RA patients (n = 578) for 12 weeks with fenebrutinib did not show increased bleeding events, also at higher dosages (150 mg QD, 200 mg BIS) which were required for clinical efficacy [49]. Additionally, in a recent summary of the results of a trial in SLE patients (n = 260) treated with the same high doses of fenebrutinib for 48 weeks no bleeding was noted [112]. Plasma Cmax of 600 nM reported after intake of 150 mg BID by human volunteers [111] are 12-fold higher than the concentrations which suppress completely Btk-dependent platelet aggregation in blood (50 nM) [13] (Table 2). Due to the lack of effect of fenebrutinib on Tec kinase [79] bleeding is not to be expected (see Section 4.3). In support even very high concentrations of fenebrutinib (up to 1 µM) did not increase bleeding time in vitro [13] (Table 2).

Additionally, for the reversible covalent rilzabrutinib, which shows positive data in trials of pemphigus and ITP, increased bleeding events have not been reported despite of very low platelet counts of ITP patients [116] (see Section 3.4) This might be explained by the absence of a drug effect on platelets by the applied dosage (see Section 3.4).

## 5. Conclusions

There are large differences between the BTKi regarding their mode of action, potencies, kinase selectivity, PD and PK properties, targeted disease, and bleeding side effects. Patients with B-cell malignancies treated with ibrutinib and the 2nd generation irreversible BTKi acalabrutinib, zanubrutinib and tirabrutinib show a high incidence of grade 1 and 2 bleeding events. It is likely that therapeutic concentrations of these BTKi ibrutinib, acalabrutinib, zanubrutinib and tirabrutinib inhibit in platelets irreversibly Tec in addition to Btk and thus strongly suppress GPVI signaling. Although strong GPVI inhibition per se might not cause bleeding, hemorrhage might occur in combination with other factors (i.e., thrombocytopenia, inflammation, co-treatment with antiplatelet drugs and anticoagulants). The low frequency of minor bleeding events of patients with PCNSL treated with tirabrutinib underlines the role of the systemic manifestation of B-cell malignancies in causing bleeding.

Branebrutinib, a very potent irreversible covalent BTKi with special PK and PD properties and off-target inhibition of Tec, is at present in a RA trial. No bleeding events were reported in a phase 1 study by participants who received branebrutinib at increasing dosage for 14 days (Section 3.1.5). In contrast, the recent phase 1 study of healthy volunteers with the novel very potent BTKi BI 705564 which also inhibits Tec showed mild bleeding-events, increased skin bleeding time and PFA closure time (Section 3.1.6). Thus, the reason for the different effects on bleeding in healthy volunteers by these two irreversible BTKi is unlikely to be off-target Tec inhibition. 

Remibrutinib, a very potent irreversible covalent BTKi, is highly selective for Btk and barely inhibits Tec. Its effects on platelets are unknown. No PK and PD data in human volunteers are available. Based on the reported PD properties in blood, remibrutinib is expected to inhibit Btk-mediated platelet signaling in whole blood with high potency. Remibrutinib is currently in phase 2 clinical studies for chronic spontaneous urticaria (CSU) and Sjögren syndrome.

Evobrutinib and tolebrutinib are 2nd generation irreversible BTKi with off-target inhibition of Tec. For evobrutinib, no bleeding has been reported in two clinical trials (MS, RA). Evobrutinib inhibits in vitro similar to the other irreversible BTKi GPIb-, GPVI- and FcγRIIa-stimulated platelet aggregation in blood, albeit at high micromolar concentrations, which can, however, be explained with its low potency to inhibit Btk. Since no PK and PD data are available in humans, no firm conclusions can be drawn of possible platelet effects in vivo after application of therapeutic concentrations of evobrutinib. It can be hypothesized that the absence of bleeding events in the two trials may be due to the different diseases targeted (MS, RA) 

Patients treated with reversible BTKi (BMS-986142, fenebrutinib) did not show bleeding events in clinical trials. Since BMS-986142 in contrast to fenebrutinib has off-target activity on Tec, it seems that a possible reversible platelet Tec inhibition may not cause bleeding side effects. Fenebrutinib might be considered as safer, since it did not show grade 1–2 bleeding events in the CLL trial, and even at very high concentrations did not increase bleeding in vitro (Section 3.3.2).

Summing up, for many BTKi which are or will be in clinical trials their effects on platelets are not known. It is not clear, whether off-target inhibition of Tec as found in in vitro kinase assays might be related to bleeding. A recent study shows that BTKi have differential impact on the conformational state of full-length Btk [141]. Whereas ibrutinib binding had long-range allosteric effects on the SH2-and SH3- regulatory domains changing their conformation towards an activated state of the protein, fenebrutinib and another covalent irreversible BTKi (CC-292, spebrutinib) did not show these conformational changes. It can be hypothesized that the BTKi which affect the conformation of regulatory domains of Btk will enable the protein to interact with and inhibit other proteins/ enzymes important for platelet responses that prevent bleeding. In support, fenebrutinib does not elicit bleeding, and in a phase 1 study of patients with CLL treated with spebrutinib, only 8% of patients showed mild bleeding events [142]. This BTKi was not followed up further due to its inferior clinical activity in B-cell malignancies.

The development of future BTKi might take into consideration their possible effects on platelets, and include studies of platelet aggregation (MEA) and closure time (PFA) in blood (as described in this review, such as Table 2) after prolonged exposure to therapeutic concentrations of BTKi in vitro and/or after oral intake ex vivo. Inhibition of platelet aggregation could be exploited for possible therapeutic applications of BTKi (atherothrombosis, venous thrombosis, HIT; Section 2), whereas the measurement of closure time might help to estimate their probability to elicit bleeding side effects in vivo.

## Figures and Tables

**Table 1:** Bruton-tyrosine kinase inhibitors (BTKi) approved or in clinical trials. Mode of inhibition, targeted disease, bleeding side effects, and platelet-relevant off-target kinase activity.

Generic Name	Brand Name	First Designation	Mode of Inhibition	Disease	Development Stage	Bleeding	Off-Target
**Irreversible BTKi**							
Ibrutinib	Imbruvica^®^	PCI-32765	Covalent (Cys-481)	CCL MCL WM MZL GVHD	Approved (2013) Approved (2017)	39% (any grade), 4% ≥ grade 3, 0.4% fatal Bleeding (>20%)	Tec, Src-kinases (Src, Lyn, Fyn, Yes) Itk
				GVHD	Approved (2017)	Bleeding (>20%)	Itk
Acalabrutinib	Calquence^®^	ACP-160	Covalent (Cys-481)	MCL CCL	Approved (2017) FDA	22% * (any grade), 3% ≥ grade 3, 0.1% fatal	Tec
Zanubrutinib	Brukinsa^®^	BGB-3111	Covalent (Cys-481)	MCL	Approved (2019) FDA	50% (any grade) 2% ≥ grade 3	Tec
Tirabrutinib	Velexbru^®^	ONO/GS-4059	Covalent (Cys-481)	PCNSL LPL WM	Approved (3/2020) Japan	Yes, see text	Tec
Branebrutinib		BMS-986195	Covalent (Cys-481)	SLE, Sjögren syndrome	Phase 1 Phase 2	No Unknown	Tec
		BI 705564	Covalent (Cys-481)	SLE, RA	Phase 1	15% (grade 1,2)	Tec
Remibrutinib		LOU064	Highly selective, Covalent (Cys-481)	CSU, Sjögren syndrome	Phase 2	Not known	no
**Irreversible BTKi, brain-penetrant**							
Evobrutinib		M2951	Covalent (Cys-481)	MS	Phase 1,2 Phase 3 **	no	Tec
Tolebrutinib		SAR- 442168, PRN2246	Covalent (Cys-481)	MS	Preclinical, Phase 1	no	Tec
**Reversible BTKi**							
		BMS-986142	reversible	RA	Phase 1,2	no	Tec
Fenebrutinib		GDC-0853	reversible	NHL,CLL RA SLE	Phase 1 Phase 2 Phase 2	no no no	no
Rilzabrutinib		PRN1008	reversible, transient covalent (Cys-481)	ITP Pemphigus	Phase 2 Phase 2	no no	Tec

* excluding petechiae and bruising, ** phase 3 started in 2020. CLL, chronic lymphocytic leukemia, identical with SLL, small lymphocytic lymphoma; CSU, chronic spontaneous urticaria; cGVHD, chronic graft versus host disease; Itk, interleukin-2 inducible kinase; ITP, diopathic thrombocytopenic purpura; LPL, lymphoplasmacytic lymphoma; MCL, mantle cell lymphoma; MS, multiple sclerosis; MZL, marginal zone lymphoma; NHL, non-Hodgkin lymphoma; PCNSL, primary central nervous system lymphoma; RA, rheumatoid arthritis; SLE, systemic lupus erythematosus; Tec, tyrosine kinase expressed in hepatocellular carcinoma; WM, Waldenström’s macroglobulinemia.

**Table 2:** Btk-inhibitors (BTKi): Comparison of IC50 values for inhibition of Btk-dependent platelet aggregation (PA) by GPVI or FcγRIIa stimulation of blood, therapeutic plasma concentrations and blood concentrations which increase bleeding time in vitro.

BTKi	Dosage	GPVI—Mediated PA *, IC50, µM	FcγRIIa-Mediated PA ** IC50, µM	Plasma Concentration (Cmax)	Increased Bleeding Time In Vitro ***		Ref.
					µM	fold ****	
**Irreversible BTKi**							
Ibrutinib	420 mg QD	0.025	0.08	0.31	1	40	13, 41, 75
Acalabrutinib	100 mg BID	0.372	0.38	1.78	5	13	13, 41, 78
Zanubrutinib	160 mg BID	0.094	0.11	1.4	nd		13, 41, 86
Tirabrutinib	480 mg QD	0.268	0.42	2.36	5	18	13, 41, 91
Evobrutinib	75 mg QD 75 mg BID	1.2	1.13	not known	nd		13, 41
**Reversible BTKi**							
Fenebrutinib	150 mg BID	0.013	0.011	0.6	no		13, 111

* Hirudin-anticoagulated blood was pre-incubated with the BTKi for 60 min before stimulation with low collagen concentrations (0.2–0-5 µg/mL) inducing the same degree of submaximal platelet aggregation as maximal concentrations of atherosclerotic plaque homogenate or with plaque homogenate (after fenebrutinib pre-incubation). Platelet aggregation was measured in blood by multiple electrode aggregometry (MEA) [42,43]. ** Hirudin-anticoagulated blood was pre-incubated with the BTKi for 60 min before CD32 cross-linking. ***, closure time was measured with the platelet-function analyzer (PFA; collagen/epinephrine cartridge) [48]. ****, x-fold over the IC50 for inhibition of GPVI-mediated aggregation. **nd:** not detected, no increase of closure time was observed by testing less than 10-fold higher concentrations of zanubrutinib (1 µM) and evobrutinib (10 µM). Higher concentrations were not tested. **no**: no increase of closure time was observed by testing up to 78,000-fold higher concentrations of fenebrutinib.

## Data Availability

Not applicable. No data are provided in this review.

## References

[1] Pal Singh S., Dammeijer F., Hendriks R.W. (2018). Role of Bruton’s tyrosine kinase in B cells and malignancies. Mol. Cancer.

[2] Whang J.A., Chang B.Y. (2014). Bruton’s tyrosine kinase inhibitors for the treatment of rheumatoid arthritis. Drug Discov. Today.

[3] Chang B.Y., Huang M.M., Francesco M., Chen J., Sokolove J., Magadala P., Robinson W.H., Buggy J.J. (2011). The Bruton tyrosine kinase inhibitor PCI-32765 ameliorates autoimmune arthritis by inhibition of multiple effector cells. Arthritis Res. Ther..

[4] Liang C., Tian D., Ren X., Ding S., Jia M., Xin M., Thareja S. (2018). The development of Bruton’s tyrosine kinase (BTK) inhibitors from 2012 to 2017: A mini-review. Eur. J. Med. Chem..

[5] Rigg R.A., Aslan J.E., Healy L.D., Wallisch M., Thierheimer M.L., Loren C.P., Pang J., Hinds M.T., Gruber A., McCarty O.J. (2015). Oral administration of Bruton’s Tyrosine Kinase (Btk) inhibitors impairs GPVI-mediated platelet function. Am. J. Physiol. Cell Physiol..

[6] Futatani T., Watanabe C., Baba Y., Tsukada S., Ochs H.D. (2001). Bruton’s tyrosine kinase is present in normal platelets and its absence identifies patients with X-linked agammaglobulinaemia and carrier females. Br. J. Haematol..

[7] Shillitoe B., Gennery A. (2017). X-Linked Agammaglobulinaemia: Outcomes in the modern era. Clin. Immunol..

[8] Busygina K., Jamasbi J., Seiler T., Deckmyn H., Weber C., Brandl R., Lorenz R., Siess W. (2018). Oral Bruton tyrosine kinase inhibitors selectively block atherosclerotic plaque-triggered thrombus formation in humans. Blood.

[9] Busygina K., Denzinger V., Bernlochner I., Weber C., Lorenz R., Siess W. (2019). Btk inhibitors as first oral atherothrombosis- selective antiplatelet drugs?. Thromb. Haemost..

[10] Nicolson P.L.R., Nock S.H., Hinds J., Garcia-Quintanilla L., Smith C.W., Campos J., Brill A., Pike J.A., Khan A.O., Poulter N.S. (2020). Low dose Btk inhibitors selectively block platelet activation by CLEC-2. Haematologica.

[11] Payne H., Ponomaryov T., Watson S.P., Brill A. (2017). Mice with a deficiency in CLEC-2 are protected against deep vein thrombosis. Blood.

[12] Nicolson P.L., Welsh J.D., Chauhan A., Thomas M.R., Kahn M.L., Watson S.P. (2020). A rationale for blocking thromboinflammation in COVID-19 with Btk inhibitors. Platelets.

[13] Goldmann L., Duan R., Kragh T., Wittmann G., Weber C., Lorenz R., von Hundelshausen P., Spannagl M., Siess W. (2019). Oral Bruton tyrosine kinase inhibitors block activation of the platelet Fc receptor CD32a (FcγRIIA): A new option in HIT?. Blood Adv..

[14] Siess W., Hundelshausen P.V., Lorenz R. (2020). Selective inhibition of thromboinflammation in COVID-19 by Btk inhibitors. Platelets.

[15] Treon S.P., Castillo J.J., Skarbnik A.P., Soumerai J.D., Ghobrial I.M., Guerrera M.L., Meid K., Yang G. (2020). The BTK inhibitor ibrutinib may protect against pulmonary injury in COVID-19-infected patients. Blood.

[16] Roschewski M., Lionakis M.S., Sharman J.P., Roswarski J., Goy A., Monticelli M.A., Roshon M., Wrzesinski S.H., Desai J.V., Zarakas M.A. (2020). Inhibition of Bruton tyrosine kinase in patients with severe COVID-19. Sci. Immunol..

[17] AstraZeneca Update on CALAVI Phase II Trials for Calquence in Patients Hospitalised with Respiratory Symptoms of COVID-19. https://www.astrazeneca.com/media-centre/press-releases/2020/update-on-calavi-phase-ii-trials-for-calquence-in-patients-hospitalised-with-respiratory-symptoms-of-covid-19.html.

[18] Watson S.P., Herbert J.M., Pollitt A.Y. (2010). GPVI and CLEC-2 in hemostasis and vascular integrity. J. Thromb. Haemost..

[19] Moroi A.J., Watson S.P. (2015). Impact of the PI3-kinase/Akt pathway on ITAM and hemITAM receptors: Haemostasis, platelet activation and antithrombotic therapy. Biochem. Pharm..

[20] Arman M., Krauel K. (2015). Human platelet IgG Fc receptor FcgammaRIIA in immunity and thrombosis. J. Thromb. Haemost..

[21] Liu J., Fitzgerald M.E., Berndt M.C., Jackson C.W., Gartner T.K. (2006). Bruton tyrosine kinase is essential for botrocetin/VWF-induced signaling and GPIb-dependent thrombus formation in vivo. Blood.

[22] Mohamed A.J., Yu L., Backesjo C.M., Vargas L., Faryal R., Aints A., Christensson B., Berglof A., Vihinen M., Nore B.F. (2009). Bruton’s tyrosine kinase (Btk): Function, regulation, and transformation with special emphasis on the PH domain. Immunol. Rev..

[23] Duarte D.P., Lamontanara A.J., La Sala G., Jeong S., Sohn Y.K., Panjkovich A., Georgeon S., Kükenshöner T., Marcaida M.J., Pojer F. (2020). Btk SH2-kinase interface is critical for allosteric kinase activation and its targeting inhibits B-cell neoplasms. Nat. Commun..

[24] Watson S.P., Auger J.M., McCarty O.J., Pearce A.C. (2005). GPVI and integrin alphaIIb beta3 signaling in platelets. J. Thromb. Haemost..

[25] Baba Y., Hashimoto S., Matsushita M., Watanabe D., Kishimoto T., Kurosaki T., Tsukada S. (2001). BLNK mediates Syk-dependent Btk activation. Proc. Natl. Acad. Sci. USA.

[26] Gibbins J.M. (2004). Platelet adhesion signalling and the regulation of thrombus formation. J. Cell Sci..

[27] Siess W. (1989). Molecular mechanisms of platelet activation. Physiol. Rev..

[28] Pasquet J.M., Quek L., Stevens C., Bobe R., Huber M., Duronio V., Krystal G., Watson S.P. (2000). Phosphatidylinositol 3,4,5-trisphosphate regulates Ca(2+) entry via btk in platelets and megakaryocytes without increasing phospholipase C activity. Embo J..

[29] Tomlinson M.G., Woods D.B., McMahon M., Wahl M.I., Witte O.N., Kurosaki T., Bolen J.B., Johnston J.A. (2001). A conditional form of Bruton’s tyrosine kinase is sufficient to activate multiple downstream signaling pathways via PLC Gamma 2 in B cells. BMC Immunol..

[30] Takata M., Kurosaki T. (1996). A role for Bruton’s tyrosine kinase in B cell antigen receptor-mediated activation of phospholipase C-gamma 2. J. Exp. Med..

[31] Nicolson P.L.R., Hughes C.E., Watson S., Nock S.H., Hardy A.T., Watson C.N., Montague S.J., Malcor J.D., Thomas M.R., Pollitt A.Y. (2018). Inhibition of Btk by Btk-specific concentrations of ibrutinib and acalabrutinib delays but does not block platelet aggregation to GPVI. Haematologica.

[32] Zeiler M., Moser M., Mann M. (2014). Copy number analysis of the murine platelet proteome spanning the complete abundance range. Mol. Cell. Proteom. MCP.

[33] Burkhart J.M., Vaudel M., Gambaryan S., Radau S., Walter U., Martens L., Geiger J., Sickmann A., Zahedi R.P. (2012). The first comprehensive and quantitative analysis of human platelet protein composition allows the comparative analysis of structural and functional pathways. Blood.

[34] Oda A., Ikeda Y., Ochs H.D., Druker B.J., Ozaki K., Handa M., Ariga T., Sakiyama Y., Witte O.N., Wahl M.I. (2000). Rapid tyrosine phosphorylation and activation of Bruton’s tyrosine/Tec kinases in platelets induced by collagen binding or CD32 cross-linking. Blood.

[35] Atkinson B.T., Ellmeier W., Watson S.P. (2003). Tec regulates platelet activation by GPVI in the absence of Btk. Blood.

[36] Quek L.S., Bolen J., Watson S.P. (1998). A role for Bruton’s tyrosine kinase (Btk) in platelet activation by collagen. Curr. Biol. CB.

[37] Kamel S., Horton L., Ysebaert L., Levade M., Burbury K., Tan S., Cole-Sinclair M., Reynolds J., Filshie R., Schischka S. (2015). Ibrutinib inhibits collagen-mediated but not ADP-mediated platelet aggregation. Leukemia.

[38] Levade M., David E., Garcia C., Laurent P.A., Cadot S., Michallet A.S., Bordet J.C., Tam C., Sie P., Ysebaert L. (2014). Ibrutinib treatment affects collagen and von Willebrand factor-dependent platelet functions. Blood.

[39] Bye A.P., Unsworth A.J., Desborough M.J., Hildyard C.A.T., Appleby N., Bruce D., Kriek N., Nock S.H., Sage T., Hughes C.E. (2017). Severe platelet dysfunction in NHL patients receiving ibrutinib is absent in patients receiving acalabrutinib. Blood Adv..

[40] Chen J., Kinoshita T., Gururaja T., Sukbuntherng J., James D., Lu D., Whang J., Versele M., Chang B.Y. (2018). The effect of Bruton’s tyrosine kinase (BTK) inhibitors on collagen-induced platelet aggregation, BTK, and tyrosine kinase expressed in hepatocellular carcinoma (TEC). Eur. J. Haematol..

[41] Denzinger V., Busygina K., Jamasbi J., Pekrul I., Spannagl M., Weber C., Lorenz R., Siess W. (2019). Optimizing platelet GPVI inhibition versus hemostatic impairment by ibrutinib and the novel Btk-inhibitors acalabrutinib, ONO/GS-4059, BGB-3111 and evobrutinib. Thromb. Haemost..

[42] Toth O., Calatzis A., Penz S., Losonczy H., Siess W. (2006). Multiple electrode aggregometry: A new device to measure platelet aggregation in whole blood. Thromb. Haemost..

[43] Bampalis V.G., Brantl S.A., Siess W. (2012). Why and how to eliminate spontaneous platelet aggregation in blood measured by multiple electrode aggregometry. J. Thromb. Haemost..

[44] Kodama M., Yamasaki Y., Sakamoto K., Yoshioka R., Matsuhisa M., Kajimoto Y., Kosugi K., Ueda N., Hori M. (2000). Antiplatelet drugs attenuate progression of carotid intima-media thickness in subjects with type 2 diabetes. Thromb. Res..

[45] Bye A.P., Gibbins J.M. (2018). Move along, nothing to see here: Btk inhibitors stop platelets sticking to plaques. J. Thromb. Haemost..

[46] Payrastre B., Ribes A. (2021). Low-dose Btk inhibitors: An ‘aspirin’ of tomorrow?. Haematologica.

[47] Kratzer M.A., Negrescu E.V., Hirai A., Yeo Y.K., Franke P., Siess W. (1995). The Thrombostat system. A useful method to test antiplatelet drugs and diets. Semin. Thromb. Hemost..

[48] Kundu S.K., Heilmann E.J., Sio R., Garcia C., Davidson R.M., Ostgaard R.A. (1995). Description of an in vitro platelet function analyzer--PFA-100. Semin. Thromb. Hemost..

[49] Cohen S., Tuckwell K., Katsumoto T.R., Zhao R., Galanter J., Lee C., Rae J., Toth B., Ramamoorthi N., Hackney J.A. (2020). Fenebrutinib versus Placebo or Adalimumab in Rheumatoid Arthritis: A Randomized, Double-Blind, Phase II Trial (ANDES Study). Arthritis Rheumatol..

[50] Jamasbi J., Ayabe K., Goto S., Nieswandt B., Peter K., Siess W. (2017). Platelet receptors as therapeutic targets: Past, present and future. Thromb. Haemost..

[51] Gruner S., Prostredna M., Aktas B., Moers A., Schulte V., Krieg T., Offermanns S., Eckes B., Nieswandt B. (2004). Anti-glycoprotein VI treatment severely compromises hemostasis in mice with reduced alpha2beta1 levels or concomitant aspirin therapy. Circulation.

[52] Lee R.H., Piatt R., Conley P.B., Bergmeier W. (2017). Effects of ibrutinib treatment on murine platelet function during inflammation and in primary hemostasis. Haematologica.

[53] Ohlmann P., Hechler B., Ravanat C., Loyau S., Herrenschmidt N., Wanert F., Jandrot-Perrus M., Gachet C. (2008). Ex vivo inhibition of thrombus formation by an anti-glycoprotein VI Fab fragment in non-human primates without modification of glycoprotein VI expression. J. Thromb. Haemost..

[54] Reny J.L., De Moerloose P., Dauzat M., Fontana P. (2008). Use of the PFA-100™ closure time to predict cardiovascular events in aspirin-treated cardiovascular patients: A systematic review and meta-analysis. J. Thromb. Haemost..

[55] Rayes J., Watson S.P., Nieswandt B. (2019). Functional significance of the platelet immune receptors GPVI and CLEC-2. J. Clin. Investig..

[56] Hitchcock J.R., Cook C.N., Bobat S., Ross E.A., Flores-Langarica A., Lowe K.L., Khan M., Dominguez-Medina C.C., Lax S., Carvalho-Gaspar M. (2015). Inflammation drives thrombosis after Salmonella infection via CLEC-2 on platelets. J. Clin. Investig..

[57] Kazianka L., Drucker C., Skrabs C., Thomas W., Melchardt T., Struve S., Bergmann M., Staber P.B., Porpaczy E., Einberger C. (2017). Ristocetin-induced platelet aggregation for monitoring of bleeding tendency in CLL treated with ibrutinib. Leukemia.

[58] Alberelli M.A., Innocenti I., Autore F., Laurenti L., De Candia E. (2018). Ibrutinib does not affect ristocetin-induced platelet aggregation evaluated by light transmission aggregometry in chronic lymphocytic leukemia patients. Haematologica.

[59] Ninomoto J., Mokatrin A., Kinoshita T., Marimpietri C., Barrett T.D., Chang B.Y., Sukbuntherng J., James D.F., Crowther M. (2020). Effects of ibrutinib on in vitro platelet aggregation in blood samples from healthy donors and donors with platelet dysfunction. Hematology.

[60] Hsu J., Gu Y., Tan S.L., Narula S., DeMartino J.A., Liao C. (2013). Bruton’s Tyrosine Kinase mediates platelet receptor-induced generation of microparticles: A potential mechanism for amplification of inflammatory responses in rheumatoid arthritis synovial joints. Immunol. Lett..

[61] Ankri A., Baranger A., Martin-Toutain I., Samson Y., Collet J.P., Montalescot G., Lejean L. (2011). Impaired ristocetin–induced Platelet Aggregation in Whole Blood Assessed with a Multiplate© Analyser Suggests a New Mechanism of Antiplatelet Effect of Aspirin and Clopidogrel. Blood.

[62] Kovacevic K.D., Greisenegger S., Langer A., Gelbenegger G., Buchtele N., Pabinger I., Petroczi K., Zhu S., Gilbert J.C., Jilma B. (2021). The aptamer BT200 blocks von Willebrand factor and platelet function in blood of stroke patients. Sci. Rep..

[63] Veyradier A. (2020). A new drug for an old concept: Aptamer to von Willebrand factor for prevention of arterial and microvascular thrombosis. Haematologica.

[64] Qiao J., Al-Tamimi M., Baker R.I., Andrews R.K., Gardiner E.E. (2015). The platelet Fc receptor, FcgammaRIIa. Immunol. Rev..

[65] Zufferey A., Kapur R., Semple J.W. (2017). Pathogenesis and Therapeutic Mechanisms in Immune Thrombocytopenia (ITP). J. Clin. Med..

[66] Miltiadous O., Hou M., Bussel J.B. (2020). Identifying and treating refractory ITP: Difficulty in diagnosis and role of combination treatment. Blood.

[67] Wei G., Luo Q., Wang X., Wu X., Xu M., Ding N., Zhao Y., Zhong L., Wang J., Wu Y. (2019). Increased GPIbα shedding from platelets treated with immune thrombocytopenia plasma. Int. Immunopharmacol..

[68] Reiff S.D., Muhowski E.M., Guinn D., Lehman A., Fabian C.A., Cheney C., Mantel R., Smith L., Johnson A.J., Young W.B. (2018). Noncovalent inhibition of C481S Bruton tyrosine kinase by GDC-0853: A new treatment strategy for ibrutinib-resistant CLL. Blood.

[69] Byrd J.C., Smith S., Wagner-Johnston N., Sharman J., Chen A.I., Advani R., Augustson B., Marlton P., Renee Commerford S., Okrah K. (2018). First-in-human phase 1 study of the BTK inhibitor GDC-0853 in relapsed or refractory B-cell NHL and CLL. Oncotarget.

[70] Bond D.A., Woyach J.A. (2019). Targeting BTK in CLL: Beyond Ibrutinib. Curr. Hematol. Malig. Rep..

[71] Sunesis Pharmaceuticals Sunesis Pharmaceuticals Announces Clinical Update on Vecabrutinib Program. https://www.globenewswire.com/news-release/2020/06/23/2051898/0/en/Sunesis-Pharmaceuticals-Announces-Clinical-Update-on-Vecabrutinib-Program.html.

[72] IMBRUVICA Highlights of Prescribing Information. https://imbruvica.com/files/prescribing-information.pdf.

[73] Jaglowski S.M., Blazar B.R. (2018). How ibrutinib, a B-cell malignancy drug, became an FDA-approved second-line therapy for steroid-resistant chronic GVHD. Blood Adv..

[74] Pharmacyclics, Janssen Biotech IMBRUVICA®—The Only Approved Oral, Once-Daily Treatment for Previously Treated Chronic Graft Versus Host Disease (cGVHD). https://imbruvicahcp.com/cgvhd.

[75] Advani R.H., Buggy J.J., Sharman J.P., Smith S.M., Boyd T.E., Grant B., Kolibaba K.S., Furman R.R., Rodriguez S., Chang B.Y. (2013). Bruton tyrosine kinase inhibitor ibrutinib (PCI-32765) has significant activity in patients with relapsed/refractory B-cell malignancies. J. Clin. Oncol. Off. J. Am. Soc. Clin. Oncol..

[76] De Vries R., Smit J.W., Hellemans P., Jiao J., Murphy J., Skee D., Snoeys J., Sukbuntherng J., Vliegen M., de Zwart L. (2016). Stable isotope-labelled intravenous microdose for absolute bioavailability and effect of grapefruit juice on ibrutinib in healthy adults. Br. J. Clin. Pharmacol..

[77] De Jong J., Skee D., Murphy J., Sukbuntherng J., Hellemans P., Smit J., de Vries R., Jiao J.J., Snoeys J., Mannaert E. (2015). Effect of CYP3A perpetrators on ibrutinib exposure in healthy participants. Pharmacol. Res. Perspect..

[78] Byrd J.C., Harrington B., O’Brien S., Jones J.A., Schuh A., Devereux S., Chaves J., Wierda W.G., Awan F.T., Brown J.R. (2016). Acalabrutinib (ACP-196) in Relapsed Chronic Lymphocytic Leukemia. N. Engl. J. Med..

[79] Crawford J.J., Johnson A.R., Misner D.L., Belmont L.D., Castanedo G., Choy R., Coraggio M., Dong L., Eigenbrot C., Erickson R. (2018). Discovery of GDC-0853: A Potent, Selective, and Noncovalent Bruton’s Tyrosine Kinase Inhibitor in Early Clinical Development. J. Med. Chem..

[80] Senis Y.A., Mazharian A., Mori J. (2014). Src family kinases: At the forefront of platelet activation. Blood.

[81] Bye A.P., Unsworth A.J., Vaiyapuri S., Stainer A.R., Fry M.J., Gibbins J.M. (2015). Ibrutinib Inhibits Platelet Integrin alphaIIbbeta3 Outside-In Signaling and Thrombus Stability But Not Adhesion to Collagen. Arter. Thromb Vasc Biol.

[82] CALQUENCE (2020). Highlights of Prescribing Information. https://www.azpicentral.com/calquence/calquence.pdf.

[83] Astrazeneca Calquence Showed Long-Term Efficacy and Tolerability for Patients with Chronic Lymphocytic Leukaemia in Two Trials. https://www.astrazeneca.com/media-centre/press-releases/2020/calquence-showed-long-term-efficacy-and-tolerability-for-patients-with-chronic-lymphocytic-leukaemia-in-two-trials.html.

[84] BRUKINSA (2020). Highlights of Prescribing Information. https://www.brukinsa.com/prescribing-information.pdf.

[85] Guo Y., Liu Y., Hu N., Yu D., Zhou C., Shi G., Zhang B., Wei M., Liu J., Luo L. (2019). Discovery of Zanubrutinib (BGB-3111), a Novel, Potent, and Selective Covalent Inhibitor of Bruton’s Tyrosine Kinase. J. Med. Chem..

[86] Tam C.S., Trotman J., Opat S., Burger J.A., Cull G., Gottlieb D., Harrup R., Johnston P.B., Marlton P., Munoz J. (2019). Phase 1 study of the selective BTK inhibitor zanubrutinib in B-cell malignancies and safety and efficacy evaluation in CLL. Blood.

[87] Dobie G., Kuriri F.A., Omar M.M.A., Alanazi F., Gazwani A.M., Tang C.P.S., Sze D.M., Handunnetti S.M., Tam C., Jackson D.E. (2019). Ibrutinib, but not zanubrutinib, induces platelet receptor shedding of GPIb-IX-V complex and integrin αIIbβ3 in mice and humans. Blood Adv..

[88] PMDA (Pharmaceuticals and Medical Devices Agency) Japan (2020). VELEXBRU (Tirabrutinib). Japanese Prescribing Information. https://www.pmda.go.jp/files/000237315.pdf.

[89] Dhillon S. (2020). Tirabrutinib: First Approval. Drugs.

[90] Liclican A., Serafini L., Xing W., Czerwieniec G., Steiner B., Wang T., Brendza K.M., Lutz J.D., Keegan K.S., Ray A.S. (2020). Biochemical characterization of tirabrutinib and other irreversible inhibitors of Bruton’s tyrosine kinase reveals differences in on—and off—target inhibition. Biochim. Et Biophys. Acta. Gen. Subj..

[91] Walter H.S., Rule S.A., Dyer M.J., Karlin L., Jones C., Cazin B., Quittet P., Shah N., Hutchinson C.V., Honda H. (2016). A phase 1 clinical trial of the selective BTK inhibitor ONO/GS-4059 in relapsed and refractory mature B-cell malignancies. Blood.

[92] Yu H., Truong H., Mitchell S.A., Liclican A., Gosink J.J., Li W., Lin J., Feng J.Y., Jürgensmeier J.M., Billin A. (2018). Homogeneous BTK Occupancy Assay for Pharmacodynamic Assessment of Tirabrutinib (GS-4059/ONO-4059) Target Engagement. Slas Discov..

[93] Series J., Ribes A., Garcia C., Souleyreau P., Bauters A., Morschhauser F., Jürgensmeier J.M., Sié P., Ysebaert L., Payrastre B. (2020). Effects of novel Btk and Syk inhibitors on platelet functions alone and in combination in vitro and in vivo. J. Thromb. Haemost..

[94] Watterson S.H., Liu Q., Beaudoin Bertrand M., Batt D.G., Li L., Pattoli M.A., Skala S., Cheng L., Obermeier M.T., Moore R. (2019). Discovery of Branebrutinib (BMS-986195): A Strategy for Identifying a Highly Potent and Selective Covalent Inhibitor Providing Rapid in Vivo Inactivation of Bruton’s Tyrosine Kinase (BTK). J. Med. Chem..

[95] Liu Q., Batt D.G., Chaudhry C., Lippy J.S., Pattoli M.A., Surti N., Xu S., Carter P.H., Burke J.R., Tino J.A. (2018). Conversion of carbazole carboxamide based reversible inhibitors of Bruton’s tyrosine kinase (BTK) into potent, selective irreversible inhibitors in the carbazole, tetrahydrocarbazole, and a new 2,3-dimethylindole series. Bioorganic Med. Chem. Lett..

[96] Catlett I.M., Nowak M., Kundu S., Zheng N., Liu A., He B., Girgis I.G., Grasela D.M. (2020). Safety, pharmacokinetics and pharmacodynamics of branebrutinib (BMS-986195), a covalent, irreversible inhibitor of Bruton’s tyrosine kinase: Randomised phase I, placebo-controlled trial in healthy participants. Br. J. Clin. Pharmacol..

[97] Litzenburger T., Steffgen J., Benediktus E., Müller F., Schultz A., Klein E., Ramanujam M., Harcken C., Gupta A., Wu J. (2020). Safety, pharmacokinetics and pharmacodynamics of BI 705564, a highly selective, covalent inhibitor of Bruton’s tyrosine kinase, in Phase I clinical trials in healthy volunteers. Br. J. Clin. Pharmacol..

[98] Caldwell R.D., Qiu H., Askew B.C., Bender A.T., Brugger N., Camps M., Dhanabal M., Dutt V., Eichhorn T., Gardberg A.S. (2019). Discovery of Evobrutinib: An Oral, Potent, and Highly Selective, Covalent Bruton’s Tyrosine Kinase (BTK) Inhibitor for the Treatment of Immunological Diseases. J. Med. Chem..

[99] Haselmayer P., Camps M., Liu-Bujalski L., Nguyen N., Morandi F., Head J., O’Mahony A., Zimmerli S.C., Bruns L., Bender A.T. (2019). Efficacy and Pharmacodynamic Modeling of the BTK Inhibitor Evobrutinib in Autoimmune Disease Models. J. Immunol..

[100] Piper L. (2020). Evobrutinib fails to improve response rate in refractory RA patients. Med. Matters.

[101] Montalban X., Arnold D.L., Weber M.S., Staikov I., Piasecka-Stryczynska K., Willmer J., Martin E.C., Dangond F., Syed S., Wolinsky J.S. (2019). Placebo-Controlled Trial of an Oral BTK Inhibitor in Multiple Sclerosis. N. Engl. J. Med..

[102] Merck Evobrutinib Relapsing Multiple Sclerosis (RMS) Update Call. https://www.merckgroup.com/investors/events-and-presentations/webcasts-and-presentations/2020/en/2020_Evobrutinib_Strategy_Update_EN.pdf.

[103] Carvalho J. (2020). Tolebrutinib Prevents Myelin Loss of in Mouse Model of MS-Like Demyelination. Sclerosis News Today.

[104] Smith P.F., Owens T.D., Langrish C.L., Xing Y., Francesco M.R., Shu J., Hartmann S., Karr D., Burns R., Quesenberry R. (2019). Phase 1 Clinical Trial of PRN2246 (SAR442168), a Covalent BTK Inhibitor Demonstrates Safety, CNSExposure and Therapeutic Levels of BTK Occupancy.

[105] Watterson S.H., De Lucca G.V., Shi Q., Langevine C.M., Liu Q., Batt D.G., Beaudoin Bertrand M., Gong H., Dai J., Yip S. (2016). Discovery of 6-Fluoro-5-(R)-(3-(S)-(8-fluoro-1-methyl-2,4-dioxo-1,2-dihydroquinazolin-3(4H)-yl)-2-methylphenyl)-2-(S)-(2-hydroxypropan-2-yl)-2,3,4,9-tetrahydro-1H-carbazole-8-carboxamide (BMS-986142): A Reversible Inhibitor of Bruton’s Tyrosine Kinase (BTK) Conformationally Constrained by Two Locked Atropisomers. J. Med. Chem..

[106] Lee S.K., Xing J., Catlett I.M., Adamczyk R., Griffies A., Liu A., Murthy B., Nowak M. (2017). Safety, pharmacokinetics, and pharmacodynamics of BMS-986142, a novel reversible BTK inhibitor, in healthy participants. Eur. J. Clin. Pharmacol..

[107] Gillooly K.M., Pulicicchio C., Pattoli M.A., Cheng L., Skala S., Heimrich E.M., McIntyre K.W., Taylor T.L., Kukral D.W., Dudhgaonkar S. (2017). Bruton’s tyrosine kinase inhibitor BMS-986142 in experimental models of rheumatoid arthritis enhances efficacy of agents representing clinical standard-of-care. PLoS ONE.

[108] Bristol-Myers Squibb (2020). Efficacy and Safety Study of BMS-986142 in Patients with Moderate to Severe Rheumatoid Arthritis. Clin. Trials Gov..

[109] Di Paolo J.A., Huang T., Balazs M., Barbosa J., Barck K.H., Bravo B.J., Carano R.A., Darrow J., Davies D.R., DeForge L.E. (2011). Specific Btk inhibition suppresses B cell- and myeloid cell-mediated arthritis. Nat. Chem. Biol..

[110] Gabizon R., London N. (2020). A Fast and Clean BTK Inhibitor. J. Med. Chem..

[111] Herman A.E., Chinn L.W., Kotwal S.G., Murray E.R., Zhao R., Florero M., Lin A., Moein A., Wang R., Bremer M. (2018). Safety, Pharmacokinetics, and Pharmacodynamics in Healthy Volunteers Treated With GDC-0853, a Selective Reversible Bruton’s Tyrosine Kinase Inhibitor. Clin. Pharmacol. Ther..

[112] Isenberg D., Furie R., Jones N.S., Guibord P., Galanter J., Lee C., Mcgregor A., Toth B., Rae J., Hwang O. (2020). Efficacy, safety, and pharmacodynamics effects of the Bruton’s tyrosine kinase inhibitor, fenebrutinib (GDC-0853), in moderate to severe systemic lupus erythematosus in a phase 2 controlled study. Ann. Rheum. Dis..

[113] Bradshaw J.M., McFarland J.M., Paavilainen V.O., Bisconte A., Tam D., Phan V.T., Romanov S., Finkle D., Shu J., Patel V. (2015). Prolonged and tunable residence time using reversible covalent kinase inhibitors. Nat. Chem. Biol..

[114] Murrell D.F., Gourlay S.G., Hill R.J., Bisconte A., Francesco M., Smith P., Karr D., Outerbridge C., Varjonen K., Goodale E.C. Development of PRN1008, a novel, reversible covalent BTK inhibitor in clinical development for pemphigus. Proceedings of the Medical Dermatology Society Annual Meeting.

[115] Smith P.F., Krishnarajah J., Nunn P.A., Hill R.J., Karr D., Tam D., Masjedizadeh M., Funk J.O., Gourlay S.G. (2017). A phase I trial of PRN1008, a novel reversible covalent inhibitor of Bruton’s tyrosine kinase, in healthy volunteers. Br. J. Clin. Pharmacol..

[116] Kuter D.J., Boccia R.V., Lee E.-J., Efraim M., Tzvetkov N., Mayer J., Trněný M., Kostal M., Hajek R., McDonald V. (2019). Phase I/II, Open-Label, Adaptive Study of Oral Bruton Tyrosine Kinase Inhibitor PRN1008 in Patients with Relapsed/Refractory Primary or Secondary Immune Thrombocytopenia. Blood.

[117] Principia Biopharm Principia Announces Positive Data from Its Phase 2 Part B Trial in Pemphigus. https://www.globenewswire.com/news-release/2020/06/12/2047632/0/en/Principia-Announces-Positive-Data-from-its-Phase-2-Part-B-Trial-in-Pemphigus.html.

[118] Langrish C.L., Bradshaw J.M., Owens T.D., Campbell R.L., Francesco M.R., Karr D.E., Murray S.K., Quesenberry R.C., Smith P.F., Taylor M.D. (2017). PRN1008, a Reversible Covalent BTK Inhibitor in Clinical Development for Immune Thrombocytopenic Purpura. Blood.

[119] Pulz R., Angst D., Dawson J., Gessier F., Gutmann S., Hersperger R., Hinniger A., Janser P., Koch G., Revesz L. (2019). Design of Potent and Selective Covalent Inhibitors of Bruton’s Tyrosine Kinase Targeting an Inactive Conformation. ACS Med. Chem. Lett..

[120] Angst D., Gessier F., Janser P., Vulpetti A., Wälchli R., Beerli C., Littlewood-Evans A., Dawson J., Nuesslein-Hildesheim B., Wieczorek G. (2020). Discovery of LOU064 (Remibrutinib), a Potent and Highly Selective Covalent Inhibitor of Bruton’s Tyrosine Kinase. J. Med. Chem..

[121] Liang Q., Chen Y., Yu K., Chen C., Zhang S., Wang A., Wang W., Wu H., Liu X., Wang B. (2017). Discovery of N-(3-(5-((3-acrylamido-4-(morpholine-4-carbonyl)phenyl)amino)-1-methyl-6-oxo-1,6-dihydropyridin-3-yl)-2-methylphenyl)-4-(tert-butyl)benzamide (CHMFL-BTK-01) as a highly selective irreversible Bruton’s tyrosine kinase (BTK) inhibitor. Eur. J. Med. Chem..

[122] ClinicalTrials.gov Open-label, Multicenter, Extension Study to Evaluate Long-term Safety and Tolerability of LOU064 in Subjects With CSU. https://www.clinicaltrials.gov/ct2/show/NCT04109313?term=LOU064&draw=2&rank=1.

[123] Shatzel J.J., Olson S.R., Tao D.L., McCarty O.J.T., Danilov A.V., DeLoughery T.G. (2017). Ibrutinib-associated bleeding: Pathogenesis, management and risk reduction strategies. J. Thromb. Haemost..

[124] Sibaud V., Beylot-Barry M., Protin C., Vigarios E., Recher C., Ysebaert L. (2020). Dermatological Toxicities of Bruton’s Tyrosine Kinase Inhibitors. Am. J. Clin. Dermatol..

[125] Brown J.R., Moslehi J., Ewer M.S., O’Brien S.M., Ghia P., Cymbalista F., Shanafelt T.D., Fraser G., Rule S., Coutre S.E. (2019). Incidence of and risk factors for major haemorrhage in patients treated with ibrutinib: An integrated analysis. Br. J. Haematol..

[126] Lipsky A.H., Farooqui M.Z., Tian X., Martyr S., Cullinane A.M., Nghiem K., Sun C., Valdez J., Niemann C.U., Herman S.E. (2015). Incidence and risk factors of bleeding-related adverse events in patients with chronic lymphocytic leukemia treated with ibrutinib. Haematologica.

[127] Wang M., Rule S., Zinzani P.L., Goy A., Casasnovas O., Smith S.D., Damaj G., Doorduijn J., Lamy T., Morschhauser F. (2018). Acalabrutinib in relapsed or refractory mantle cell lymphoma (ACE-LY-004): A single-arm, multicentre, phase 2 trial. Lancet.

[128] Rule S.A., Cartron G., Fegan C., Morschhauser F., Han L., Mitra S., Salles G., Dyer M.J.S. (2020). Long-term follow-up of patients with mantle cell lymphoma (MCL) treated with the selective Bruton’s tyrosine kinase inhibitor tirabrutinib (GS/ONO-4059). Leukemia.

[129] Walter H.S., Jayne S., Rule S.A., Cartron G., Morschhauser F., Macip S., Karlin L., Jones C., Herbaux C., Quittet P. (2017). Long-term follow-up of patients with CLL treated with the selective Bruton’s tyrosine kinase inhibitor ONO/GS-4059. Blood.

[130] Munakata W., Ando K., Hatake K., Fukuhara N., Kinoshita T., Fukuhara S., Shirasugi Y., Yokoyama M., Ichikawa S., Ohmachi K. (2019). Phase I study of tirabrutinib (ONO-4059/GS-4059) in patients with relapsed or refractory B-cell malignancies in Japan. Cancer Sci..

[131] Brown J.R., Moslehi J., O’Brien S., Ghia P., Hillmen P., Cymbalista F., Shanafelt T.D., Fraser G., Rule S., Kipps T.J. (2017). Characterization of atrial fibrillation adverse events reported in ibrutinib randomized controlled registration trials. Haematologica.

[132] Pulte D., Olson K.E., Broekman M.J., Islam N., Ballard H.S., Furman R.R., Olson A.E., Marcus A.J. (2007). CD39 activity correlates with stage and inhibits platelet reactivity in chronic lymphocytic leukemia. J. Transl. Med..

[133] Goerge T., Ho-Tin-Noe B., Carbo C., Benarafa C., Remold-O’Donnell E., Zhao B.Q., Cifuni S.M., Wagner D.D. (2008). Inflammation induces hemorrhage in thrombocytopenia. Blood.

[134] Jamasbi J., Megens R.T., Bianchini M., Munch G., Ungerer M., Faussner A., Sherman S., Walker A., Goyal P., Jung S. (2015). Differential Inhibition of Human Atherosclerotic Plaque-Induced Platelet Activation by Dimeric GPVI-Fc and Anti-GPVI Antibodies: Functional and Imaging Studies. J. Am. Coll. Cardiol..

[135] Mato A.R., Timlin C., Ujjani C., Skarbnik A., Howlett C., Banerjee R., Nabhan C., Schuster S.J. (2018). Comparable outcomes in chronic lymphocytic leukaemia (CLL) patients treated with reduced-dose ibrutinib: Results from a multi-centre study. Br. J. Haematol..

[136] Chen L.S., Bose P., Cruz N.D., Jiang Y., Wu Q., Thompson P.A., Feng S., Kroll M.H., Qiao W., Huang X. (2018). A pilot study of lower doses of ibrutinib in patients with chronic lymphocytic leukemia. Blood.

[137] Akhtar O.S., Attwood K., Lund I., Hare R., Hernandez-Ilizaliturri F.J., Torka P. (2019). Dose reductions in ibrutinib therapy are not associated with inferior outcomes in patients with chronic lymphocytic leukemia (CLL). Leuk. Lymphoma.

[138] Parikh S.A., Achenbach S.J., Call T.G., Rabe K.G., Ding W., Leis J.F., Kenderian S.S., Chanan-Khan A.A., Koehler A.B., Schwager S.M. (2020). The impact of dose modification and temporary interruption of ibrutinib on outcomes of chronic lymphocytic leukemia patients in routine clinical practice. Cancer Med..

[139] Alexander W., Davis S., Ramakrishna R., Manoharan A. (2020). Outcomes of Reduced Frequency Dosing of Ibrutinib in Chronic Lymphocytic Leukemia Patients Following Complete or Partial Remission: A Pilot Study. J. Hematol..

[140] Florian P., Wonerow P., Harder S., Kuczka K., Dubar M., Graff J. (2017). Anti-GPVI Fab SAR264565 effectively blocks GPVI function in ex vivo human platelets under arterial shear in a perfusion chamber. Eur. J. Clin. Pharmacol..

[141] Joseph R.E., Amatya N., Fulton D.B., Engen J.R., Wales T.E., Andreotti A. (2020). Differential impact of BTK active site inhibitors on the conformational state of full-length BTK. eLife.

[142] Brown J.R., Harb W.A., Hill B.T., Gabrilove J., Sharman J.P., Schreeder M.T., Barr P.M., Foran J.M., Miller T.P., Burger J.A. (2016). Phase I study of single-agent CC-292, a highly selective Bruton’s tyrosine kinase inhibitor, in relapsed/refractory chronic lymphocytic leukemia. Haematologica.

